# Railway curve squeal prediction using environmental variables

**DOI:** 10.3389/frai.2026.1887534

**Published:** 2026-08-27

**Authors:** Leevi Toratti, Praneeth Chandran, Florian Thiery, Matti Rantatalo

**Affiliations:** Division of Operation, Maintenance and Acoustics, Luleå University of Technology, Luleå, Sweden

**Keywords:** curve squeal, environmental monitoring, machine learning, predictive maintenance, railway noise

## Abstract

**Introduction:**

Curve squeal is a loud tonal noise from railway traffic that contributes to environmental noise pollution. Squealing arises from friction-induced vibrations in the wheel-rail contact during vehicle curving under specific friction conditions. Environmental factors are known to influence these friction conditions and, consequently, the tendency of squeal occurrence. This study investigates whether environmental variables measured from the wayside of the track can be used to predict curve squeal using machine learning models.

**Methods:**

Separate prediction models were developed for low-rail and high-rail squeal using a labeled dataset collected during a 19-month measurement campaign at a commuter railway curve in Sweden. Multiple machine learning algorithms were evaluated, and feature importance analysis was used to investigate the environmental predictors associated with low-rail and high-rail squeal. The practical usefulness of the prediction models was further assessed for adaptive top-of-rail friction modifier (TOR-FM) application.

**Results:**

Environmental variables contained useful predictive information regarding curve squeal occurrence, although the overall predictive performance remained limited. The tree-based ensemble models generally achieved the highest performance, while the feature importance analysis indicated that low-rail and high-rail squeal relied on different feature patterns. The operational assessment demonstrated that the prediction models could reduce unnecessary TOR-FM triggering while maintaining high squeal detection rates.

**Discussion:**

The results demonstrate the potential of combining environmental monitoring with machine learning to support site-specific predictive railway noise mitigation and maintenance decision-making. The differences between low-rail and high-rail prediction further indicate the value of treating the two squeal types as separate prediction tasks.

## 1 Introduction

Curve squeal is a tonal noise generated by wheel-rail interaction in curves, causing environmental noise issues in the vicinity of railway tracks. The wheel-rail contact experiences high creepage during curving, which can lead to friction induced stick-slip oscillations during specific contact conditions (Thompson et al., 2018). These oscillations excite the resonances of the railway wheel and initiate squeal noise.

Squeal noise may be generated on top of the rail or in the wheel flange-rail gauge corner contact. The former can be associated with the leading inner wheel that experiences high lateral creepage during curving, whereas the latter can be associated with the leading outer wheel flange rubbing against the rail gauge corner with a high normal force (Rudd, 1976). These squeal types are often distinguished by examining the character of the squeal noise, top of rail squeal having a more tonal character while flange squeal (or flange noise) having a more high frequency and broad band character (Thompson, 2009). However, in practice the squeal types can be hard to differentiate by examining recorded sound signals as they may exhibit very similar acoustic character and might be excited simultaneously. Therefore, in this study squeal events are separated into squealing generated on the low-rail (inner rail in the curve) and squealing generated on the high-rail (outer rail in the curve), without explicitly distinguishing between top of rail squeal and flange squeal.

The occurrence of curve squeal is known to vary with the environmental conditions due to changes in the wheel-rail contact conditions. In particular statistical relationships have been identified regarding temperature (Ostermann, 2021; Eriksson et al., 2024; Toratti et al., 2026), and relative humidity (Ostermann, 2021; Toratti et al., 2026; Meehan et al., 2010; Liu and Meehan, 2014; Valena et al., 2024). Also tendencies regarding daily variation have been found (Toratti et al., 2026; Venghaus, 2019). The identified relationships between squeal probability and environmental conditions suggest potential for squeal prediction using environmental features as predictors. Few studies have utilized data-driven models for squeal prediction and analysis. In Eriksson et al. (2024) a logistic regression (LR) model was utilized for investigating the tendencies of squeal occurrence regarding vehicle, track and environmental variables. The dataset consisted of 1.5 years of on-board measurements on two vehicles in the subway network of Stockholm, Sweden. Statistical trends were found regarding variables such as curve radius, air temperature and relative humidity. However, the squeal prediction performance of the model was not assessed. In Ostermann (2021) machine learning models were applied to assess feature rankings and to predict squeal occurrence using a labeled dataset consisting of wayside measurements from railway curves in Austria. Separate models were trained for predicting squeal, high-frequency squeal and flange noise. The squeal types were distinguished by analyzing the acoustic signals. Neural networks were applied for the former two squeal types, while flexible discriminant analysis was used in the latter. The features of the model included vehicle, track, operational and environmental variables. The classification performance of the models resulted in 68%–84% sensitivity and 55%–68% accuracy (or 27%–66% sensitivity and 67%–83% accuracy by varying the decision threshold).

Data-driven curve squeal prediction models, such as the ones mentioned above, enable predictive noise control measures. As an example the application rate of top-of-rail friction modifiers (TOR-FM) for squeal mitigation could be controlled by the prediction models [for more information on friction modifiers see Thompson et al. (2018) and Eadie and Santoro (2006)]. In addition, these models can give indications on which parameters and parameter values are important for curve squeal occurrence. This knowledge can be beneficial for understanding the contact conditions leading to squealing.

Advances in predictive modeling have enabled data-driven condition monitoring and predictive maintenance approaches in the railway sector. The application of these methods have been studied on a wide range of railway assets, including rail fastening systems, switches and crossings, axle bearings, and railway infrastructure, enabling fault detection, health assessment, remaining useful life estimation, and maintenance decision support (Chandran et al., 2021; Davari et al., 2021; Konecny et al., 2026; Fernandes et al., 2025). Despite these developments, the application of data-driven condition monitoring and predictive analytics for railway noise, and specifically curve squeal, remains largely unexplored.

Although previous studies have demonstrated statistical relationships between curve squeal occurrence and environmental conditions, and some have explored data-driven prediction methods, important gaps remain. First, existing studies have generally not considered squeal occurrence as a rail dependent phenomenon while squeal generated on the low-rail and high-rail may arise from different wheel–rail contact conditions and mechanisms. Consequently, it remains unclear whether environmental variables influence low-rail and high-rail squeal in a similar manner and whether separate prediction models are needed. Second, previous studies have primarily focused on identifying statistical relationships between environmental variables and squeal occurrence, whereas the practical capability of environmental measurements to predict squeal events has received less attention. Third, the relative importance of environmental predictors for low-rail and high-rail squeal prediction has not been investigated.

In this study machine learning models are developed and evaluated for predicting railway curve squeal occurrence using environmental variables measured from the wayside of the track. Separate prediction models are developed for low-rail and high-rail squeal events, enabling rail specific assessment of predictive performance and feature importance. The models are trained and validated using a labeled dataset collected during a 19-month measurement campaign at a railway (RF) curve in Sweden. Multiple machine learning algorithms are compared, including Gaussian Naive Bayes (GNB), Logistic Regression, Random Forest and Extreme Gradient Boosting (XGB). In addition, SHAP feature importance analysis is used to investigate how environmental variables contribute to model predictions.

The study builds on findings of Toratti et al. (2026), which investigated statistical relationships between environmental conditions and curve squeal occurrence. The present study explores whether environmental variables can be used to predict future squeal events using machine learning. In addition, feature importance analysis is used to investigate the contribution of individual features to model predictions, and the practical usefulness of the models is assessed in the context of adaptive TOR-FM application strategies. An operational assessment is further conducted to quantify the trade-off between squeal detection and TOR-FM application rate.

The main contributions of this work are: (1) the development of separate machine learning models for low-rail and high-rail squeal prediction, (2) assessment of the predictive value of environmental variables for rail-specific squeal occurrence, and (3) a machine learning based analysis revealing differences in the environmental predictors for low-rail and high-rail squeal. The results provide a proof-of-concept demonstration of site-specific squeal prediction using environmental variables and thus contribute to the development of predictive noise mitigation strategies for railway operations.

## 2 Methods

### 2.1 Dataset

#### 2.1.1 Data collection

A labeled dataset was used for training the squeal prediction models. The dataset consists of recorded train pass-bys from a railway curve that generates squealing noise in the Stockholm region, Sweden. The dataset is an extended version of the dataset collected in Toratti et al. (2026), with the data collection period extended from 12 months (24,903 data points) to 19 months (41,460 data points). A summary of the data collection procedure is explained in this subsection, while a detailed explanation can be found in Toratti et al. (2026).

**Figure 1:**
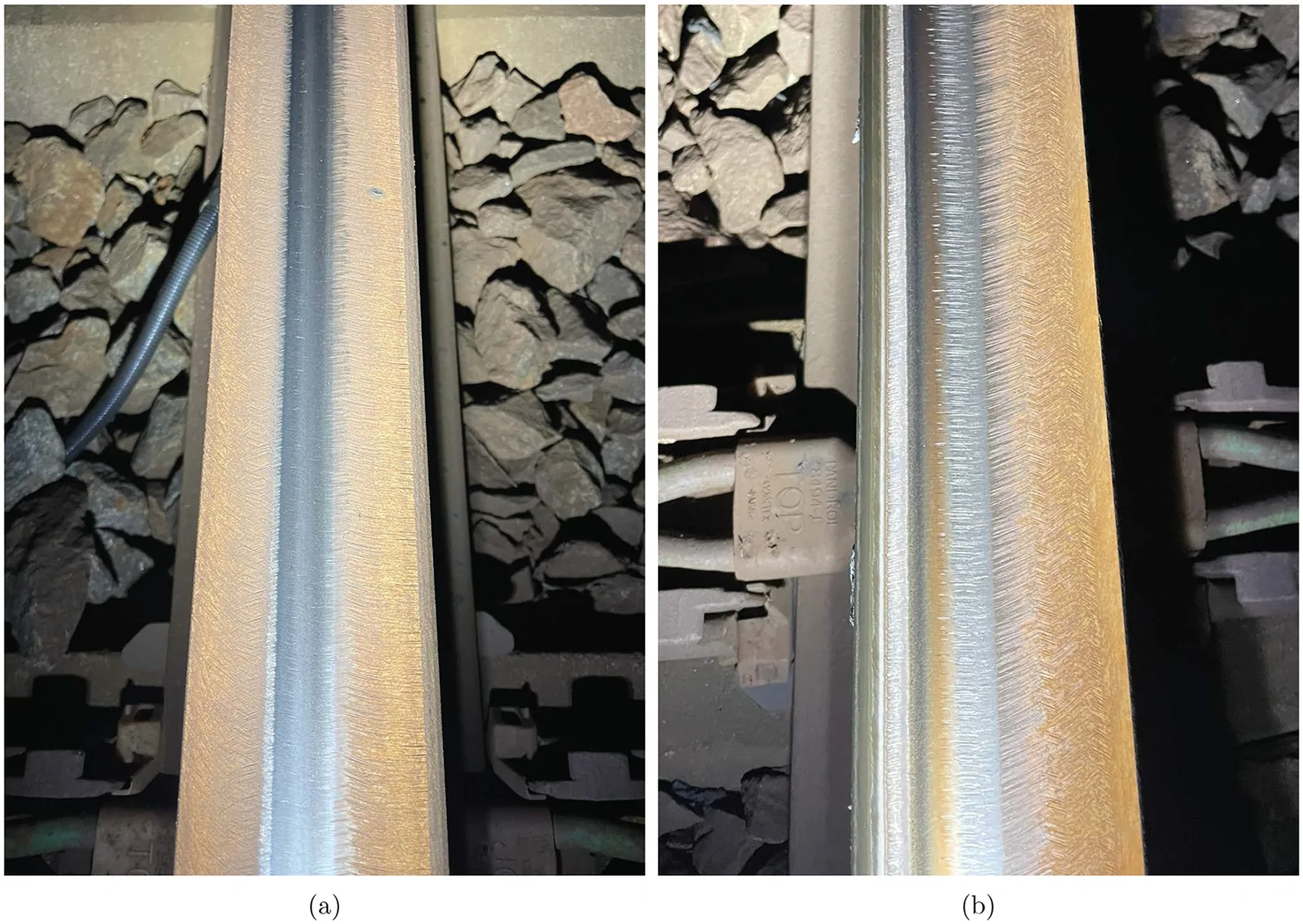
Railhead wear patterns: **(a)** low-rail and **(b)** high-rail. Figures obtained from Toratti et al. (2026).

The measurements at the curve were carried out over a 19 month period from March 27th 2024 to November 5th 2025. The circular section of the curve has a radius of 315 meters with 140 mm track cant, resulting in balanced curving at speed 60 km/h. Apart from occasional maintenance vehicles, the curve was operated by a single type of commuter train. The wear pattern on the low-rail in the curve (Figure 1a) is on top of the rail, indicating that the leading and trailing inner wheels have a contact between wheel tread and the top of the rail. The high-rail (Figure 1b) wear pattern exhibits contact zones on top of the rail and the rail gauge corner. The former is likely from the trailing outer wheel contact and the latter from the leading outer wheel contact that can have a single or two-point contact.

**Figure 2:**
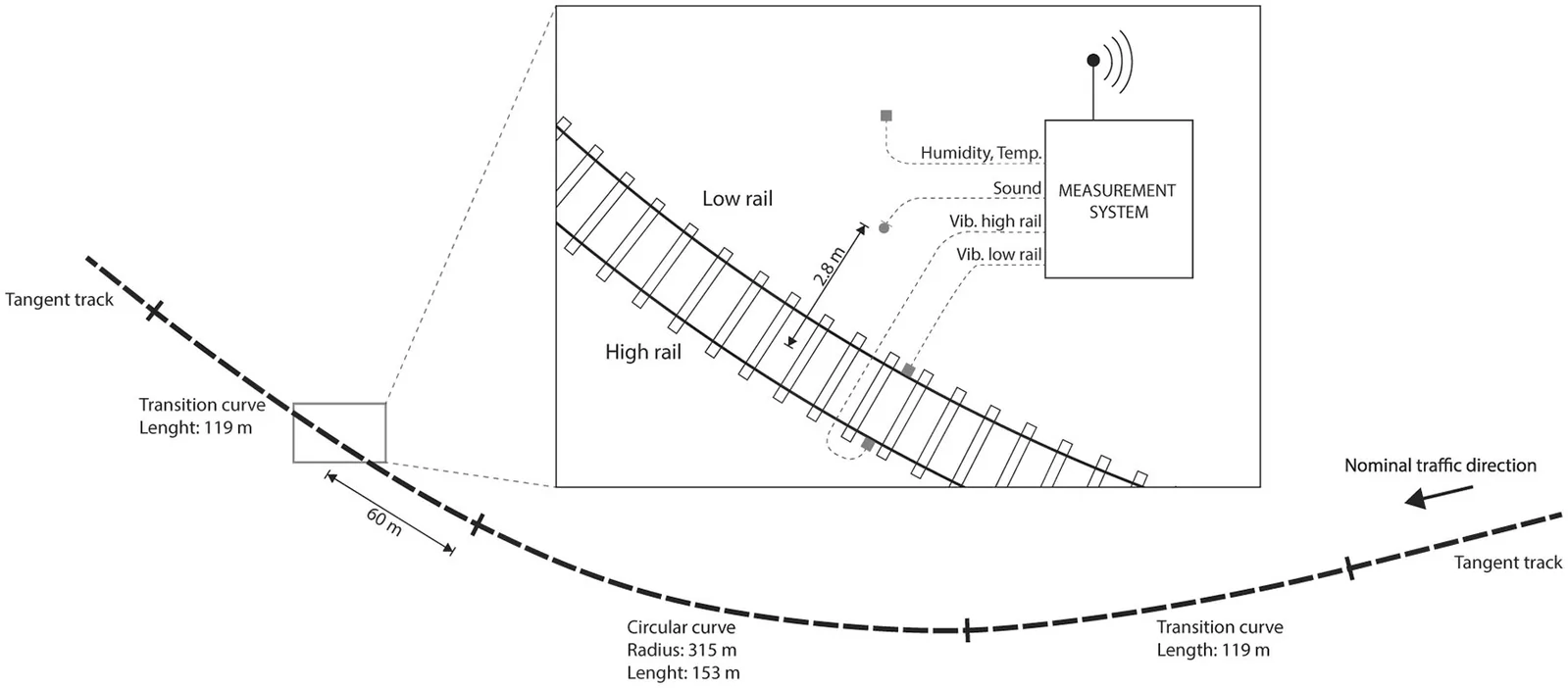
Wayside measurement system. Figure obtained from Toratti et al. (2026).

The measurement system was installed in the downstream side transition curve considering nominal traffic direction (Figure 2). The system recorded the sound and rail vibration signals of each passing train. Rail vibrations were measured with single-axis accelerometers mounted laterally on the rail web of the low- and high-rail. Sound was measured with a microphone 2.8 meters from the track middle and 0.5 meters above the rail head. Furthermore, during each measurement, environmental variables were recorded using a weather station and a rail temperature sensor.

Detection of curve squeal was done by analyzing the rail vibration signals and the sound signal in parallel, enabling the distinction between squeal events on the low-rail and high-rail. A squeal event was denoted whenever two conditions were met: (1) the rail vibration signal exceeded predefined thresholds for a sustained time period and (2) the sound signal contained one or more tonal components in the frequency range 1-10 kHz during the same time period. A detailed description of the algorithm for squeal detection is presented in Toratti et al. (2026).

#### 2.1.2 Dataset composition

Data quality control was performed prior to compiling the final dataset. A total of 861 data points were excluded during data handling due to unreliable sensor readings. These observations were mostly associated with rail temperatures below 0 °C and affected specific months of the measurement campaign: March 2024, April 2024, September 2024, October 2024, November 2024, and December 2024. Furthermore, 12 days of measurements in April 2024 and 9 days in June 2024 were lost due to technical flaws in data gathering.

The final measurement dataset contained 41,460 data points in total. Each data point corresponds to a single train pass-by event, represented by a collection of measured and derived features, along with a binary label indicating the presence or absence of a squeal event. Specifically, positive class (1) denotes that the pass-by contained squealing, and negative class (0) indicates that no squealing was detected.

Within the dataset, squealing on the low-rail was observed in approximately 11% of the samples (4,740 data points) and squealing on the high-rail in approximately 27% of the samples (11,004 data points). In approximately 4% of the samples (1,559 data points) squeal events were observed both on the low-rail and high-rail.

To enable independent prediction of squeal occurrence on each rail, two distinct versions of the dataset were constructed. Both datasets share the same datapoints and features but differ in labeling:

- Dataset 1: Squeal events on the low-rail. Class distribution: {0: 89%, 1: 11%}
- Dataset 2: Squeal events on the high-rail. Class distribution: {0: 73%, 1: 27%}

#### 2.1.3 Temporal structure

**Figure 3:**
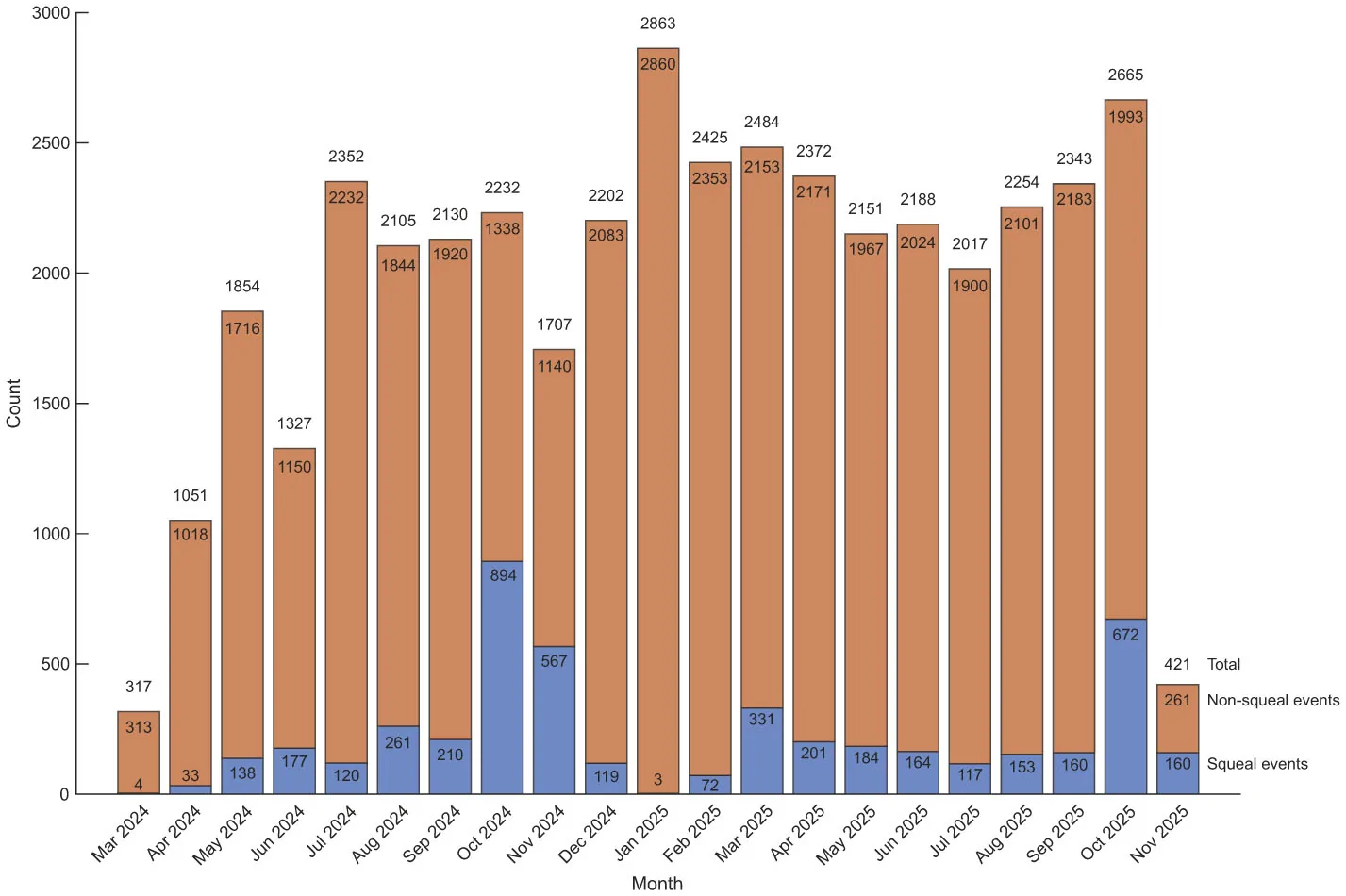
Monthly count of squeal and non-squeal events on the low-rail.

**Figure 4:**
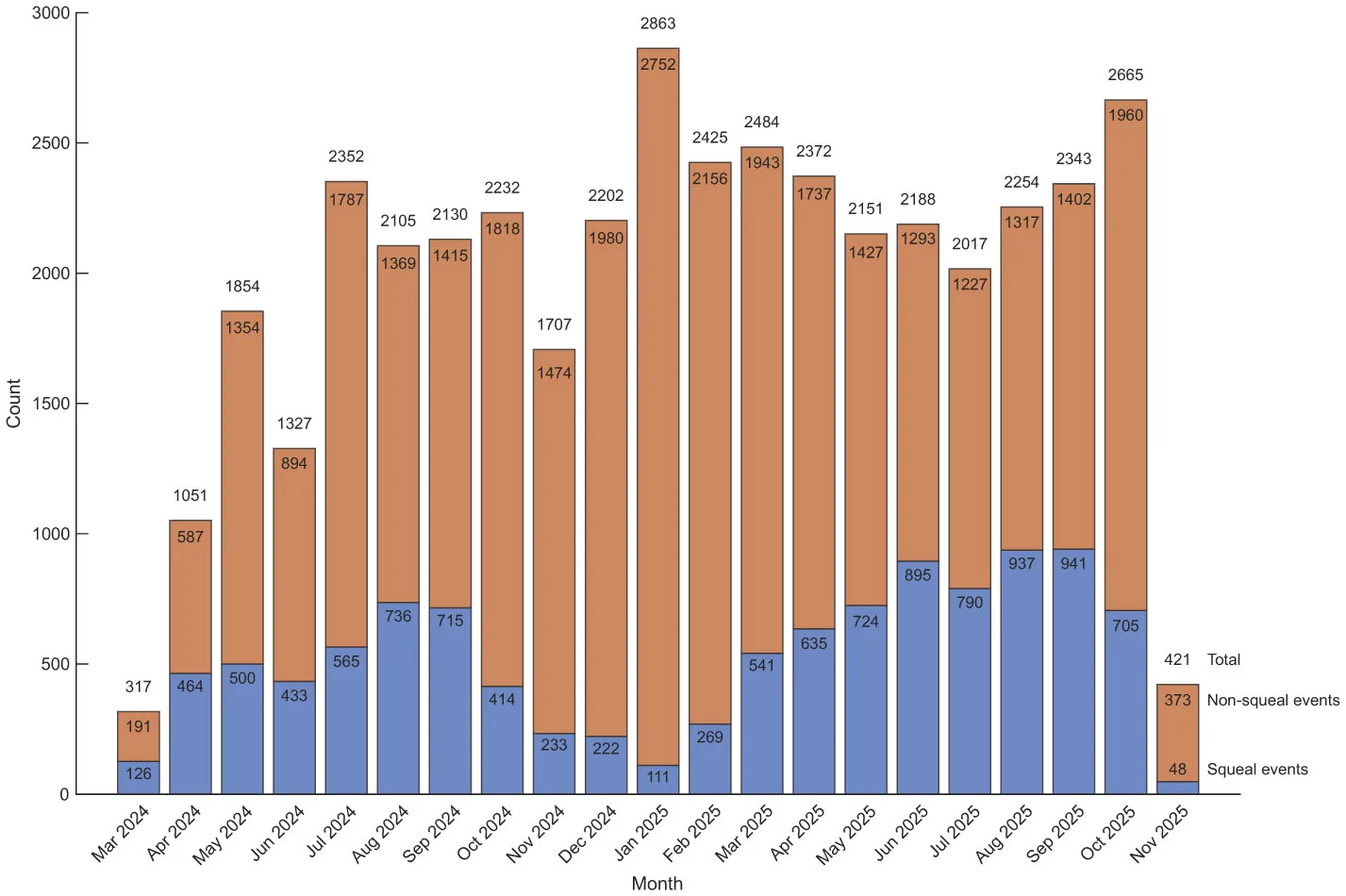
Monthly count of squeal and non-squeal events on the high-rail.

Figures 3, 4 show the temporal structure of the dataset as a bar graph of the monthly counts of squeal and non-squeal events for low-rail and high-rail. The dataset shows temporal variation in both sample size and squeal occurrence rates throughout the 19-month measurement campaign. The number of recorded train pass-bys varied between months, from 1,051 observations in April 2024 to 2,863 observations in January 2025. The significantly low counts in March 2024 and November 2025 are due to that only parts of these months were included in the dataset as they represent the start month and end month of the measurement campaign.

In addition to the variation in sample size, the proportion of squeal events also changed over time. Low-rail squeal occurrence showed seasonal variation. The monthly proportion of squeal events ranged from nearly 0% in January 2025 to nearly 40% during October 2024 (894 out of 2,232 observations) and 25% in October 2025 (672 out of 2,665 observations). Periods with high squeal occurrence rate were observed during autumn, specifically October month, whereas winter months showed notably lower squeal rates.

High-rail squeal occurrence showed also seasonal variation, although the pattern differed from that observed for the low-rail squeal. Monthly squeal rates ranged from approximately 4% in January 2025 (111 out of 2,863 observations) to over 40% during summer and early autumn 2025 (e.g., 937 out of 2,254 observations in August 2025 and 941 out of 2,343 observations in September 2025). In general, winter months exhibited lower squeal rates.

Both the class distribution and environmental conditions vary across months and seasons. Consequently, randomly distributing samples between training and test sets may allow adjacent observations with very similar environmental conditions to appear in both sets, potentially leading to too optimistic performance estimates. Therefore, time-aware validation procedures were used to assess the ability of the models to generalize to unseen future periods.

### 2.2 Features

The features of the dataset include a range of directly measured environmental parameters and additional engineered parameters that are derived from these. The features were limited to environmental variables that can be measured continuously from the wayside of the track, as the models are intended to be used for local real-time prediction. A summary of the feature explanations is covered in this subsection, while the environmental features are explained in detail in Toratti et al. (2026).

Measured features consisted of air temperature *T*_*air*_, rail temperature *T*_*rail*_, and relative air humidity *H*_*R, air*_. Dew point *T*_*dew*_ was provided as such by the weather measurement station, however, it is not directly measured but derived from *T*_*air*_ and *H*_*R, air*_ by the measurement unit.

Absolute air humidity *H*_*A, air*_ was calculated according to the formulation presented in Parish and Putnam (1977):


$$\begin{array}{l} H_{A,air}=\frac{6.113\cdot e^{\left[\frac{17.67\cdot T_{air}}{T_{air}+243.5}\right]}\cdot H_{R,air}\cdot 2.1674}{273.15+T_{air}} \end{array}$$ (1)

Relative rail humidity *H*_*R, rail*_ was estimated using rail temperature in the following equations for saturation water vapor pressure *e*_*sat*_ (Equation 2) and partial water vapor pressure *e*′ (Equation 3) in hPa, and relative humidity *H*_*R, rail*_ (Equation 4) in %. The original forms of the equations were obtained from WMO (2023):


$$\begin{array}{l} e_{sat}\left(T\right)=6.112\cdot {\text{e}}^{\frac{17.62\cdot T}{243.12+T}}, \end{array}$$ (2)

$$\begin{array}{l} e^{\prime }=\frac{H_{R,air}}{100}\cdot e_{sat}\left(T_{air}\right) \end{array}$$ (3)

and


$$\begin{array}{l} H_{R,rail}=\frac{e^{\prime }}{e_{sat}\left(T_{rail}\right)}\cdot 100. \end{array}$$ (4)

The friction coefficients were estimated according to the model presented in Galas et al. (2020). The model estimates wheel-rail adhesion coefficients for a clean contact μ_*clean*_ and a contaminated contact μ_*cont*_ based on relative air humidity *H*_*R, air*_ and air temperature *T*_*air*_ using equations


$$\begin{array}{l} \mu_{clean}=0.5461+0.003029\cdot T_{air}-0.002844\cdot H_{R,air}\\ \;-3.346\cdot 10^{-5}\cdot T_{air}^{2}+5.401\cdot 10^{-5}\cdot T_{air}\cdot H_{R,air} \end{array}$$ (5)

and


$$\begin{array}{l} \mu_{cont}=0.4153+0.001587\cdot T_{air}-0.001662\cdot H_{R,air}. \end{array}$$ (6)

**Figure 5:**
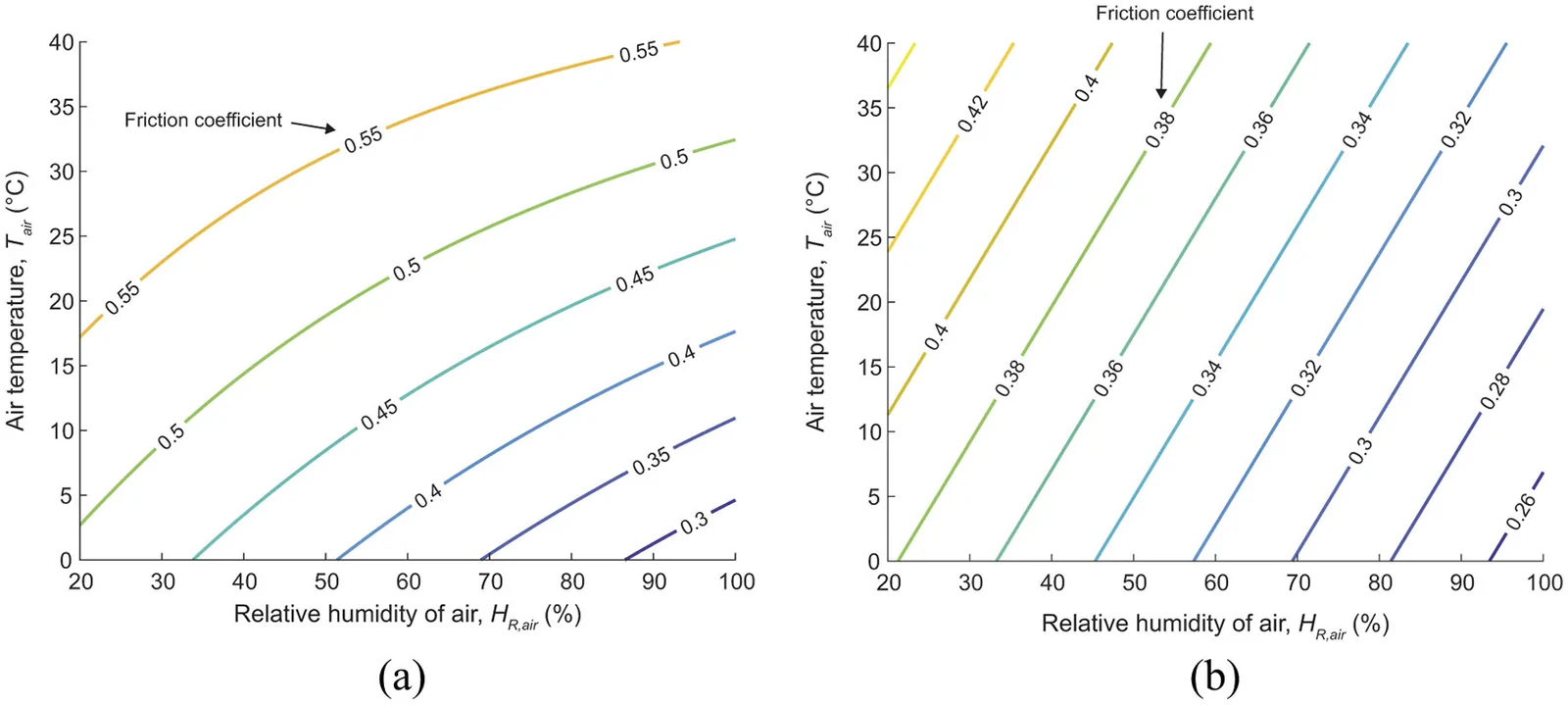
Estimated friction coefficient of **(a)** clean and **(b)** contaminated contact for varying temperature and relative humidity according to model presented in Galas et al. (2020). **(a)** Obtained from Toratti et al. (2026).

Figure 5 shows the estimated μ_*clean*_ and μ_*cont*_ with respect to *H*_*R, air*_ and *T*_*air*_. In general, the model exhibits a decreasing friction coefficient for increasing relative humidity and decreasing temperature, with a quadratic relationship for μ_*clean*_, and a linear relationship for μ_*cont*_.

Hour of day is a categorical feature consisting of discrete time bins of 1 h. Due to its cyclical nature it is represented as two features; its sine component *h*_*sin*_ and its cosine component *h*_*cos*_. These are calculated as


$$\begin{array}{l} h_{sin}=\text{sin}\left(\frac{2\pi \cdot hour}{24}\right) \end{array}$$ (7)

and


$$\begin{array}{l} h_{cos}=\text{cos}\left(\frac{2\pi \cdot hour}{24}\right). \end{array}$$ (8)

Table 1 summarizes the used features and their minimum value, maximum value, mean value and standard deviation. The feature set consists of four categories, namely directly measured variables (*T*_*air*_, *T*_*rail*_, *H*_*R, air*_, *h*_*sin*_ and *h*_*cos*_), derived humidity variables (*T*_*dew*_, *H*_*A, air*_, *H*_*R, rail*_), temperature differences (Δ*T*_*R*−*A*_, Δ*T*_*R*−*D*_, Δ*T*_*A*−*D*_) and estimated friction coefficients (μ_*clean*_, μ_*cont*_).

**Table 1:** Features and their minimum, maximum, mean value and standard deviation.

Feature	Symbol	Description	Unit	Min/Max	Mean	Std
1	*T*_*air*_	Air temperature	°C	–13.1/33.9	11.7	8.2
2	*T*_*rail*_	Rail temperature	°C	–12.4/49.7	15.4	11.3
3	*T*_*dew*_	Dew point	°C	–15.1/21.7	6.4	6.5
4	*H*_*R, air*_	Relative air humidity	%	14.5/100.0	73.1	19.5
5	*H*_*A, air*_	Absolute air humidity (Equation 1)	g/m^3^	1.6/18.9	7.9	3.2
6	*H*_*R, rail*_	Relative rail humidity (Equation 4)	%	6.6/125.2	62.7	25.1
7	Δ*T*_*R*−*A*_	Difference *T*_*rail*_−*T*_*air*_	°C	–7.8/21.0	3.6	4.1
8	Δ*T*_*R*−*D*_	Difference *T*_*rail*_−*T*_*dew*_	°C	–3.3/42.6	9.0	8.4
9	Δ*T*_*A*−*D*_	Difference *T*_*air*_−*T*_*dew*_	°C	0.0/28.6	5.4	4.8
10	μ_*clean*_	Estimated friction coefficient, clean (Equation 5)	–	0.20 / 0.58	0.41	0.08
11	μ_*cont*_	Estimated friction coefficient, contaminated (Equation 6)	–	0.25/0.43	0.31	0.04
12	*h*_*sin*_	Hour of day, sine component (Equation 7)	–	–1/1	–	–
13	*h*_*cos*_	Hour of day, cosine component (Equation 8)	–	–1/1	–	–

#### 2.2.1 Feature distributions

Histograms of the environmental features over the dataset are shown in Appendix A. The air temperature, rail temperature, and dew point exhibit broad variation, reflecting the seasonal variation over the 19-month measurement period. Air temperature ranges from −13 to 34 °C, while rail temperature spans a wider range from −12 to 50 °C, likely due to sun radiation heating the rail surface. The temperature distributions are unimodal.

Relative air humidity is concentrated at high values, with most observations above 60%. Relative rail humidity shows a bimodal tendency, with a peak at high humidity levels and a secondary peak at low humidity levels.

The temperature difference variables (Δ*T*_*R*−*A*_, Δ*T*_*R*−*D*_ and Δ*T*_*A*−*D*_) exhibit asymmetric distributions, with skewness to the right and concentration at small positive values. Since Δ*T*_*R*−*D*_ is an indicator of proximity to dew formation, this suggests that environmental conditions close to condensation occur frequently at the measurement site. The estimated friction coefficients show relatively narrow intervals with unimodal distributions.

#### 2.2.2 Temporal variation of features

**Figure 6:**
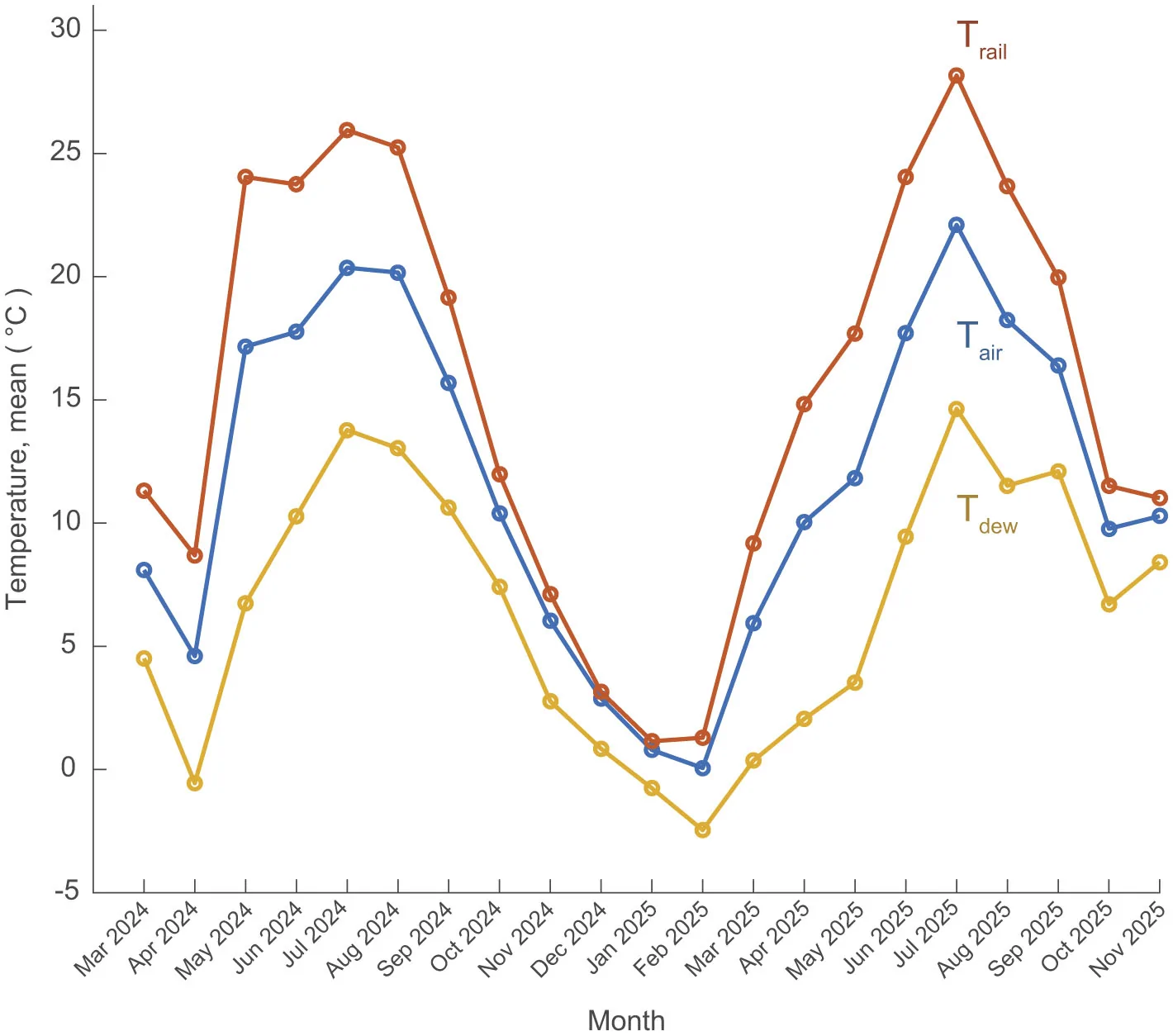
Monthly mean values of temperatures *T*_*air*_, *T*_*rail*_, and *T*_*dew*_.

**Figure 7:**
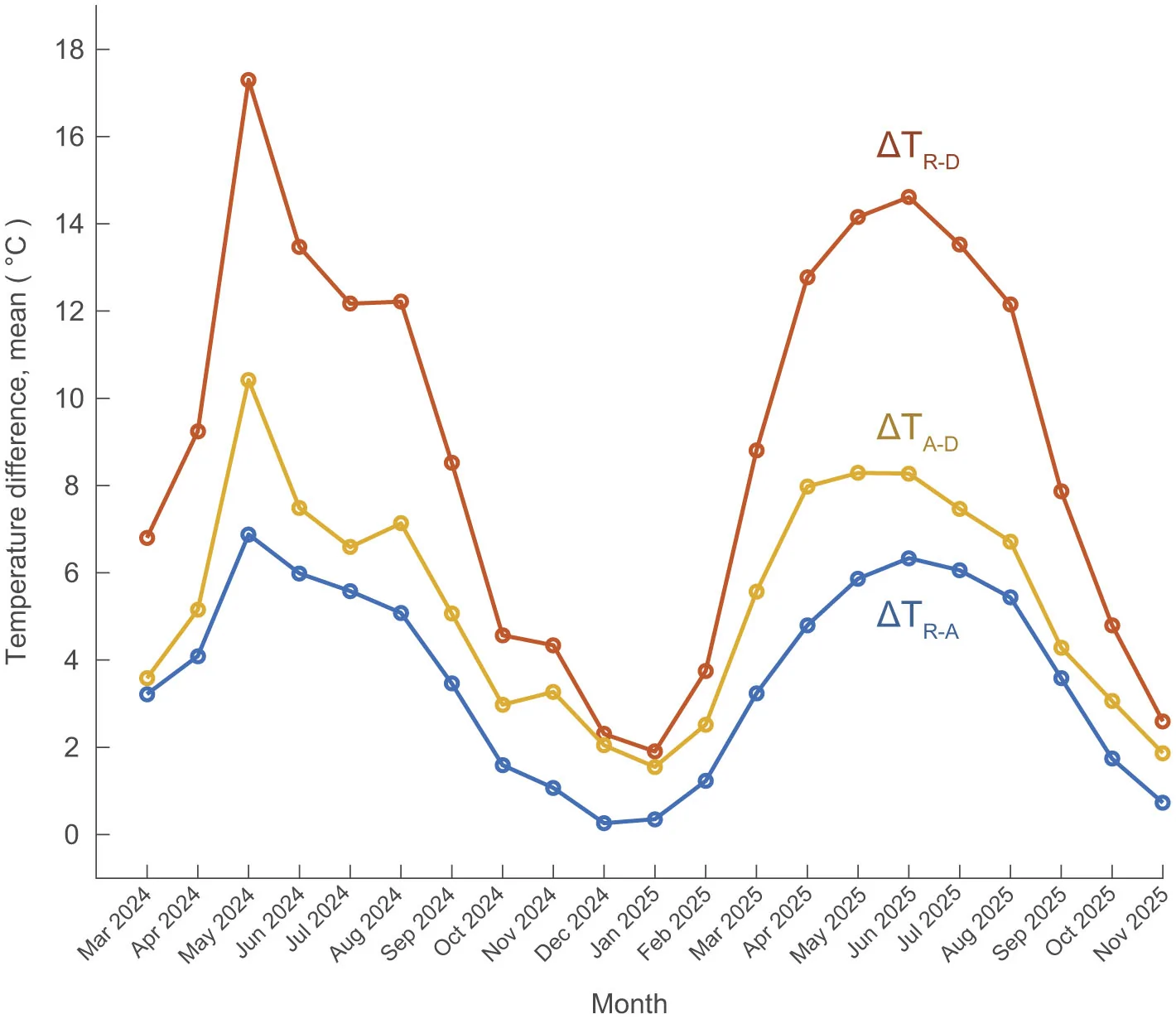
Monthly mean values of temperature differences Δ*T*_*R*−*A*_, Δ*T*_*R*−*D*_, and Δ*T*_*A*−*D*_.

**Figure 8:**
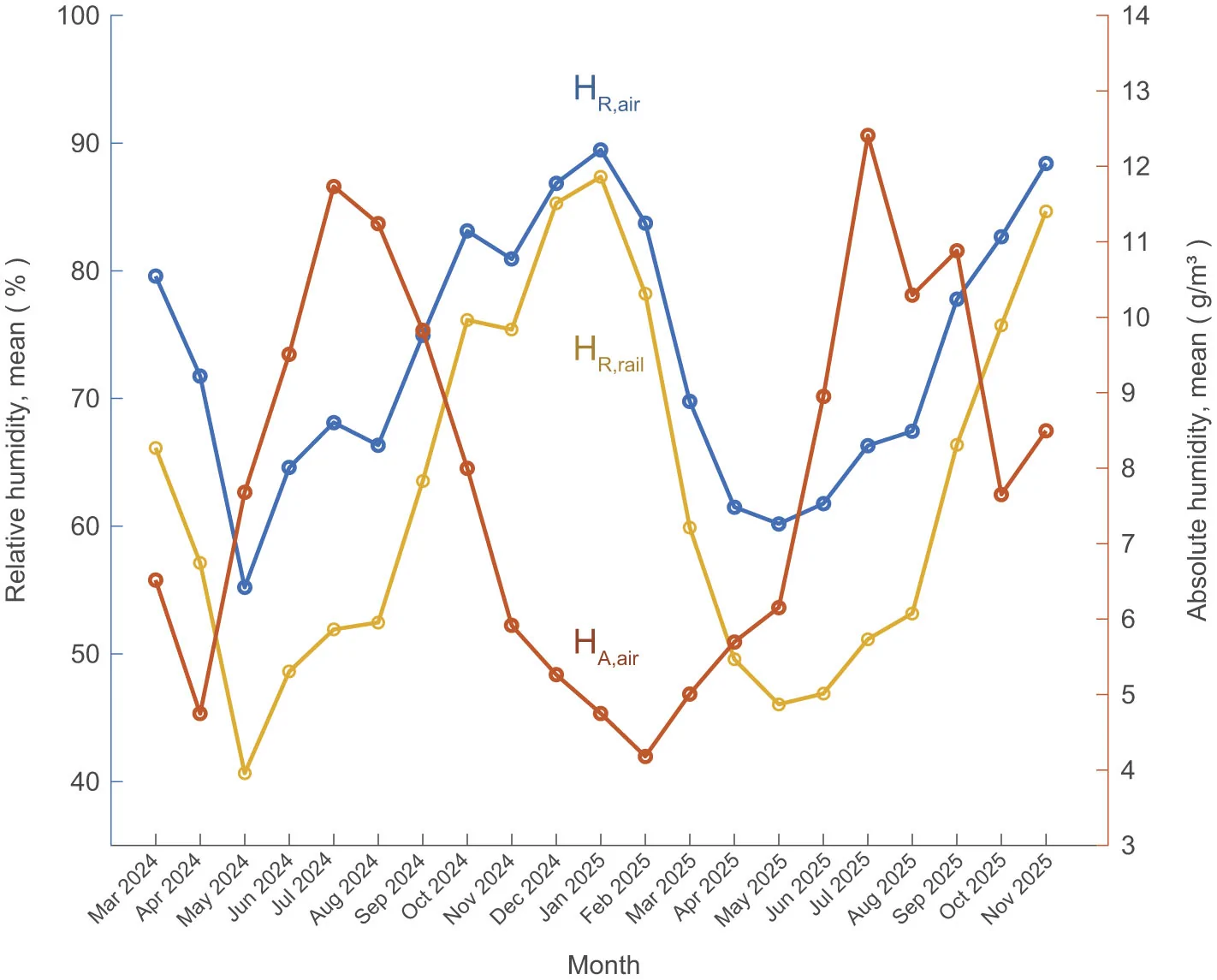
Monthly mean values of humidity features *H*_*R,air*_, *H*_*R,rail*_, and *H*_*A,air*_.

**Figure 9:**
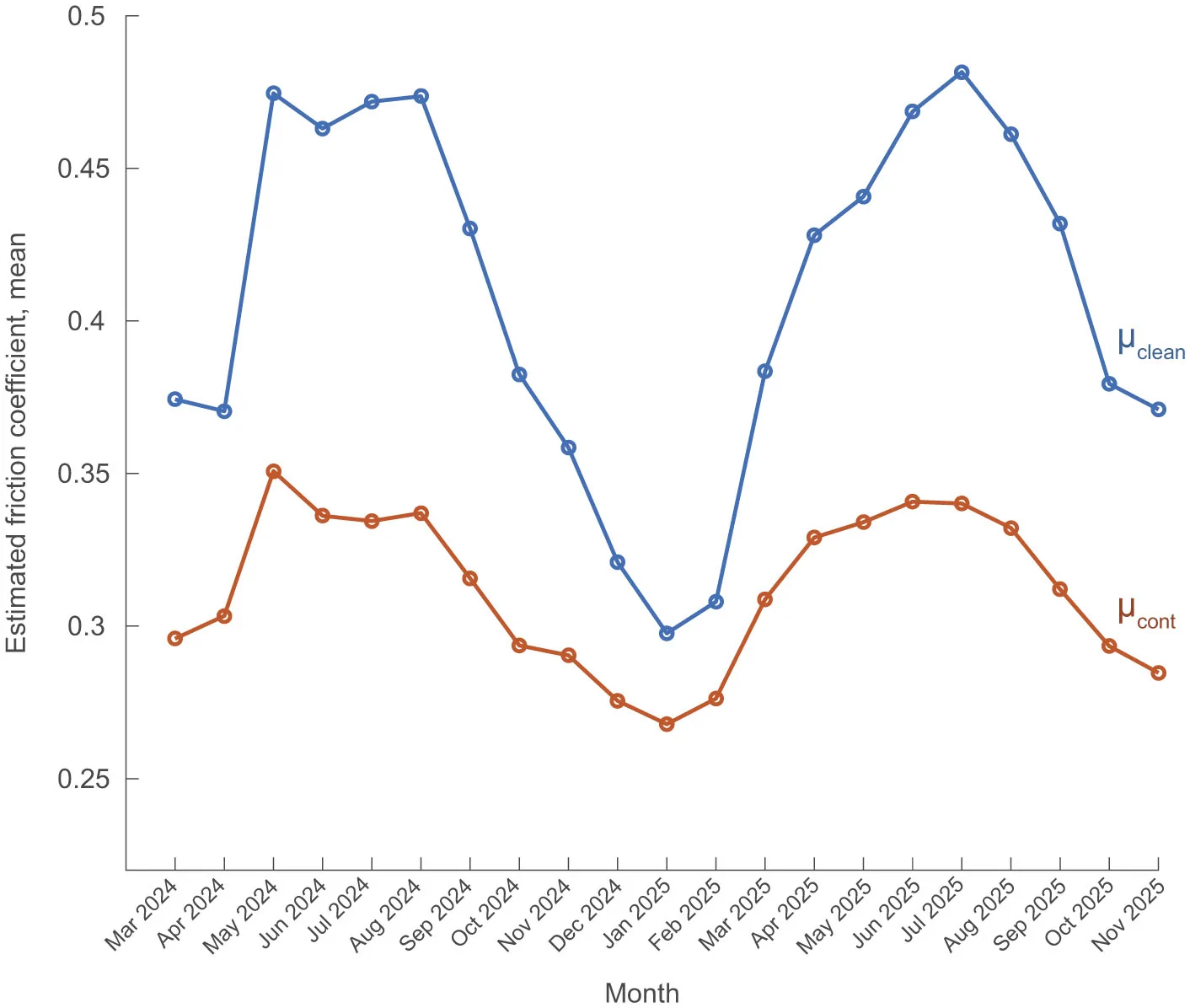
Monthly mean values of estimated friction coefficients *μ*_*clean*_ and *μ*_*cont*_.

Figures 6–9 show the monthly mean values of temperatures, temperature differences, humidity features and estimated friction coefficients, respectively. Clear temporal variation with seasonal patterns is seen for all the features throughout the measurement period. This indicates that observations collected in adjacent time periods are not independent but follow temporal patterns.

Air temperature, rail temperature, and dew point follow clear annual cycles (Figure 6), with minimum values occurring during winter (December–February) and maximum values during summer (June–August). Rail temperature show larger seasonal variation than air temperature, reflecting the influence of sun radiation heating of the rail, causing high rail temperatures during summer months. Consequently, the temperature difference features (Δ*T*_*R*−*A*_, Δ*T*_*R*−*D*_ and Δ*T*_*A*−*D*_) also show temporal variation (Figure 7), with the largest values generally occurring during summer and the smallest values during winter.

Relative humidity features show an inverse seasonal pattern compared with temperature. The highest values were observed during autumn and winter, while the lowest values occurred during summer. In contrast, absolute humidity showed a similar pattern as temperature, reaching maximum values during summer and minimum values during winter. This reflects on the airs ability to contain a higher amount of humidity at high temperatures.

The estimated friction coefficients also showed a temporal pattern (Figure 9), with seasonal cycles. The lowest values occurred during winter periods characterized by low temperatures and high humidity, whereas the highest values were observed during summer. This demonstrates that the derived estimated friction features are correlated with seasonal environmental conditions.

#### 2.2.3 Feature correlation analysis

**Figure 10:**
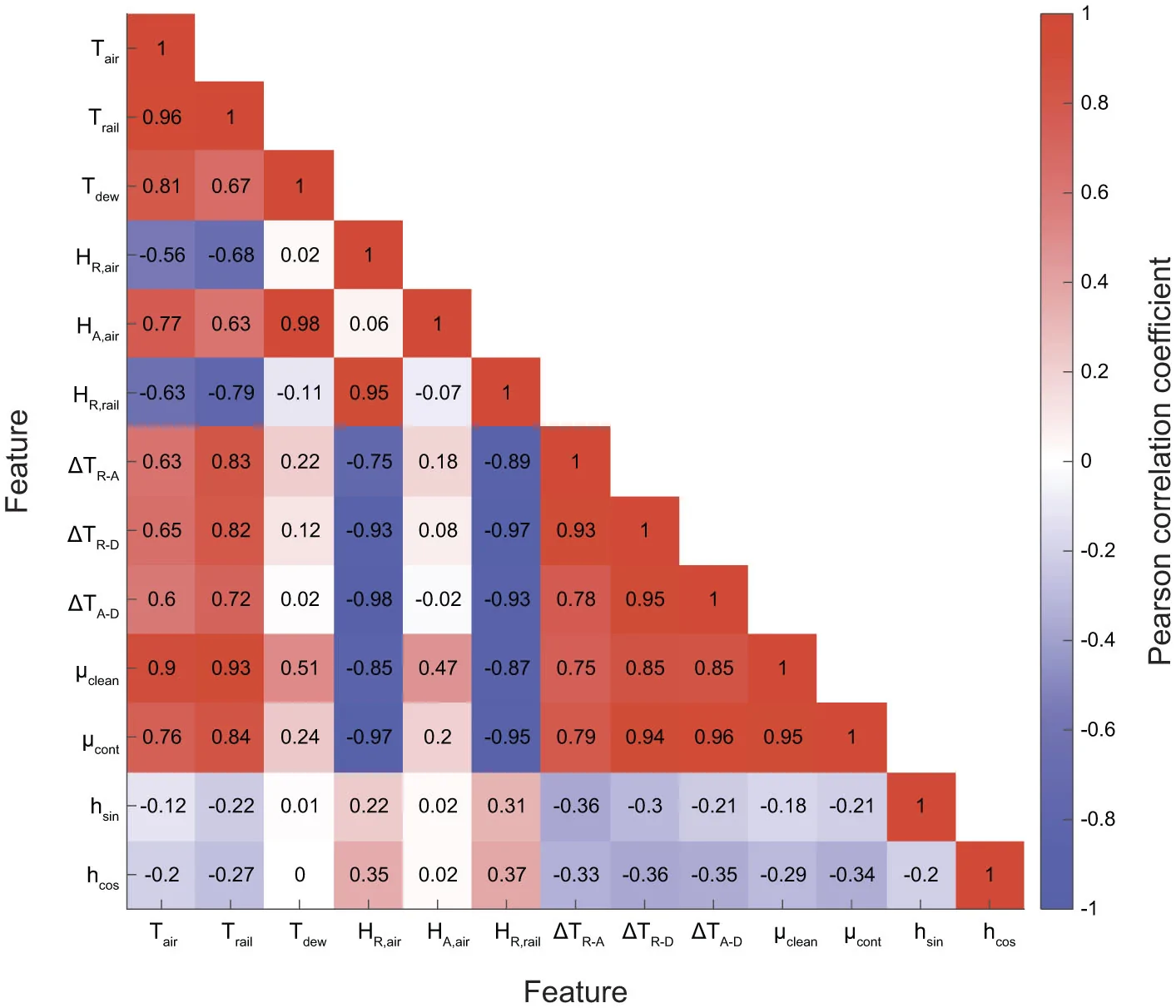
Pearson correlation matrix.

**Figure 11:**
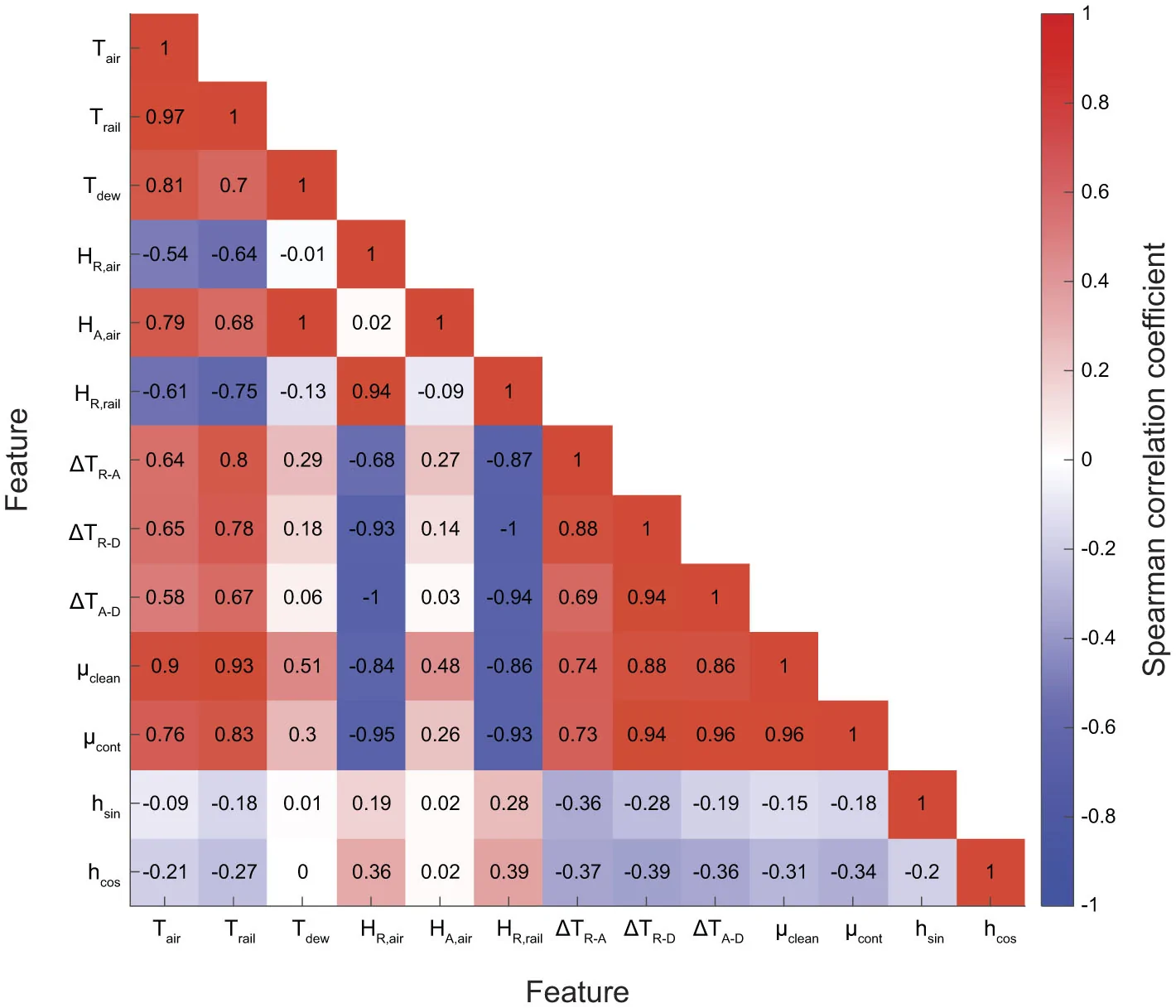
Spearman correlation matrix.

To assess correlation between features, Pearson and Spearman correlation coefficients were calculated for all features. The correlation matrices are shown in Figures 10, 11. Pearson correlation was used to evaluate linear relationships, while Spearman correlation was used to assess monotonic relationships.

The analysis showed significant dependencies among several features. Strong positive correlations were observed between air temperature and rail temperature (Pearson: 0.96, Spearman: 0.97), and the estimated friction coefficients exhibited strong correlations with temperature and humidity variables. For example, μ_*clean*_ showed strong positive correlations with air temperature (Pearson: 0.90) and rail temperature (Pearson: 0.93), and strong negative correlations with relative humidity features (Pearson: -0.85 to -0.87). Similarly, μ_*cont*_ was strongly correlated with air temperature, rail temperature, and relative humidity features.

Several derived features were also highly correlated with their source variables. For example, the temperature-difference features Δ*T*_*R*−*D*_ and Δ*T*_*A*−*D*_ showed strong correlations with relative humidity features and estimated friction coefficients. The strongest relationships were seen between *H*_*R, rail*_ and Δ*T*_*R*−*D*_ (Pearson: –0.97, Spearman: –1.00), *H*_*R, air*_ and Δ*T*_*A*−*D*_ (Pearson: –0.98, Spearman: –1.00), between the two estimated friction coefficients (Pearson: 0.95, Spearman: 0.96), and between *H*_*A, air*_ and *T*_*dew*_ (Pearson: 0.98, Spearman: 1.00). These results indicate that several features describe closely related environmental conditions and may not be statistically independent.

The Pearson and Spearman coefficients were generally similar, indicating that the relationships between features are mainly monotonic and close to linear. In contrast, the hour of day features *h*_*sin*_ and *h*_*cos*_ exhibited weak correlation with the other features, suggesting that they contain information that is generally independent of the other environmental variables.

The observed feature dependencies should be considered when interpreting feature importances. Highly correlated features may share predictive information, resulting in importance being distributed among multiple related features.

### 2.3 Prediction model

The task of predicting curve squeal occurrence is a binary classification problem. Based on a set of features (Table 1), the classifier model should output a binary value prediction indicating whether the datapoint contains squealing or not. For this task, models using different machine learning algorithms were trained with the labeled datasets and their prediction performance was assessed and compared to baseline models introduced in Subsection 2.3.6. Additionally, the feature importances of the best performing classifiers was assessed.

#### 2.3.1 Classifier algorithms

Various machine learning algorithms (Table 2) were trained with the labeled datasets. The algorithms consisted of Gaussian Naive Bayes (GNB), Logistic Regression (LR), Random Forest (RF) and Extreme Gradient Boosting (XGB). GNB represents a simple probabilistic model and LR a simple linear combination model, while RF and XGB represent more complex tree-based ensemble and boosting models, respectively. The model implementations were done with Scikit-learn machine learning library (Pedregosa et al., 2011).

**Table 2:** Classifier algorithms.

Algorithm	Abbreviation	Type	References
Gaussian Naive Bayes	GNB	Probabilistic	Pedregosa et al., 2011
Logistic regression	LR	Linear combination	Pedregosa et al., 2011
Random forest	RF	Ensemble	Breiman, 2001
Extreme gradient boosting	XGB	Boosting	Chen and Guestrin, 2016

Gaussian Naive Bayes (GNB) (Pedregosa et al., 2011) is a probabilistic classification algorithm based on Bayes' theorem. Predictions are made by calculating posterior probabilities of each class from prior probabilities and likelihood of the observed features. The predicted class is chosen as the one that obtains the highest posterior probability. GNB involves the assumptions that there are no interdependencies among features, and that the features follow a Gaussian distribution.

Logistic regression (LR) (Pedregosa et al., 2011) is a binary classification algorithm that applies a logistic (sigmoid) function to a linear combination of the feature values, transforming the output into a value between 0 and 1. During model training, the feature weights are adjusted to minimize the error function with maximum likelihood estimation. The output value represents a probability, for which a threshold is applied to in order to predict a class. Hyperparameters that were tuned for LR were the type of regularization penalty (penalty) and the penalty parameter (C) that controls the strength of regularization. The maximum number of iterations was set to 5000.

Random Forest (RF) (Breiman, 2001) is an ensemble algorithm that consists of a set of parallel decision trees. Each decision tree is trained on a random sample of the dataset, and at each node, a random subset of features is considered for splitting. The final prediction is obtained through majority vote of the individual trees. The RF models were optimized by tuning four hyperparameters: the number of decision trees (n_estimators), the maximum depth of the trees (max_depth), the minimum number of samples required for a split in a node (min_samples_split) and the minimum number of samples required at a leaf node (min_samples_leaf). These hyperparameters control overfitting and complexity of the model. Although additional hyperparameters were initially explored, they did not yield significant improvements in model performance. Thus, the tuning was limited to these parameters to reduce computational cost in model tuning.

Extreme Gradient Boosting (XGBoost, XGB) (Chen and Guestrin, 2016) is an algorithm based on gradient boosting of decision trees. XGB builds a model by sequentially constructing decision trees, where each new tree is trained to correct the residual errors of the previous ensemble using gradient descent. The tuned hyperparameters for XGB were the number of decision trees (n_estimators), the maximum depth of trees (max_depth), the learning rate that reduces the contribution of each successive tree (learning_rate) and the minimum loss reduction for performing a split in a node (gamma). Similarly to RF models, the tuning process was restricted to the aforementioned parameters, as tuning other hyperparameters did not significantly improve the model performance.

In this study the lower index in the classifier abbreviation denotes the dataset that the classifier is trained on. As an example GNB_1_ is the Gaussian Naive Bayes classifier trained on dataset 1 to predict squealing on the low-rail and XGB_2_ is the Extreme Gradient Boosting classifier trained on dataset 2 to predict squealing on the high-rail.

#### 2.3.2 Performance metrics

The model performance evaluation in this study was done by assessing how effectively the classifiers identified positive instances, corresponding to squeal events. Due to the imbalance of the classes, appropriate performance metrics were needed to assess the model performances. Metrics, such as accuracy, that do not account for the class asymmetry may not be appropriate for imbalanced datasets (Sokolova and Lapalme, 2009). The model performance in this study was assessed using the confusion matrix based metrics; precision, recall, F1-score, balanced accuracy (BA), as well as decision threshold independent metrics; precision-recall curve (PR-curve) and the average precision (AP).

The confusion matrix sorts the prediction result into classes of true negatives (TN), false negatives (FN), true positives (TP) and false positives (FP). Based on these classes a range of performance metrics can be derived.

Precision (Equation 9)


$$\begin{array}{l} Precision=\frac{TP}{TP+FP} \end{array}$$ (9)

represents the proportion of predicted positive instances that are actual positives, reflecting the reliability of positive predictions. Recall (Equation 10) (also called sensitivity or true positive rate)


$$\begin{array}{l} Recall=\frac{TP}{TP+FN} \end{array}$$ (10)

denotes the proportion of actual positive instances that were correctly identified by the classifier, highlighting the model's ability to capture the actual positive cases. Similarly, specificity (Equation 11) (also called true negative rate)


$$\begin{array}{l} Specificity=\frac{TN}{TN+FP} \end{array}$$ (11)

denotes the proportion of correctly identified negative instances out of all actual negatives.

The dataset in this study has a significant class imbalance, and therefore performance metrics that take this into account need be chosen. To assess the performance of the models with imbalanced datasets the metrics balanced accuracy (BA) (Equation 12) and F1-score (Equation 13) were chosen. BA is defined as the average of recall and specificity


$$\begin{array}{l} BA=\frac{Recall+Specificity}{2} \end{array}$$ (12)

ensuring that the performance on both negative and positive classes contribute equally to the final score. This makes BA a more appropriate metric for imbalanced datasets than standard accuracy, where the majority class is likely to dominate the evaluation. F1-score is the harmonic mean of precision and recall


$$\begin{array}{l} F1=\frac{2\cdot Precision\cdot Recall}{Precision+Recall}, \end{array}$$ (13)

whereby the class imbalance is accounted for. A high F1-score indicates that the classifier maintains a good balance between identifying true positives and avoiding false positives.

The decision threshold is a probability value between 0 and 1 that determines how the classifier distinguishes between positive and negative classes. By default this value is usually set to 0.5, but the threshold can be tuned to optimize with regard to specific performance metrics. In this study, the decision threshold was selected as the value that maximized the F1-score. This threshold was used to enable a consistent comparison between classifier models and to provide a balanced trade-off between precision and recall. However, for practical deployment, the optimal threshold may differ depending on the consequence of false positives and false negatives. Therefore, an additional operational assessment was conducted to evaluate the effect of different decision thresholds on squeal-event detection and TOR-FM application triggering.

The confusion matrix based metrics are specific to a chosen decision threshold. To assess the general model performance independent of the decision threshold, further metrics were needed. For this the chosen metrics were the PR-curve that shows the trade-off between precision and recall across all decision thresholds, and AP that provides a single scalar summary of the PR-curve by averaging the precision over all recall levels, capturing the classifier's performance across all decision thresholds. In general, a greater area under the PR-curve denotes a better performing model. An ideal model has an area under PR-curve close to 1. In such case, when choosing the decision threshold close to the upper right corner of the PR-curve, the model exhibits precision and recall close to 1. The baseline AP, corresponding to the Null classifier in this study, equals the proportion of positive instances in the dataset. This value represents the expected precision if all instances are predicted as positives.

#### 2.3.3 Training and validation

The analysis of the dataset temporal structure indicate that both the class distribution and environmental conditions vary across months and seasons. Consequently, randomly distributing samples between training and test sets may allow adjacent observations with very similar environmental conditions to appear in both sets, which can lead to unrealistically optimistic performance estimates. Therefore, chronological validation procedures were used to assess the models ability to predict future periods.

The dataset was initially split into training set and test set. The training set was used for training the classifier model while the test set was used to evaluate its performance on unseen data. All training procedures were performed using a fixed random seed of 42 to ensure reproducibility. Prior to training the LR model, the feature values were scaled to zero mean and unit variance. The rest of the models (GNB, XGB and RF) were trained using the original feature values.

The train-test split was done chronologically, with the earliest 75% of the dataset used for training set and the latest 25% used for test set. The training set covered measurements from March 2024 to June 2025, while the test set covered the remaining part of the dataset from June 2025 to November 2025. The training set had 31,095 datapoints in total out of which 3,420 (11%) were positive class in dataset 1 and 7,278 (23%) in dataset 2, while the test set had 10,365 datapoints out of which 1,320 (13%) were positive class in dataset 1 and 3,726 (36%) in dataset 2.

Hyperparameter optimization was performed on the training set using a 5-fold time series cross-validation (TimeSeriesSplit, Pedregosa et al., 2011). This method reflects the case of predicting future squeal events from historical observations. The procedure allowed for finding the optimal hyperparameters of the model that maximize the chosen performance metric during the model training process. In this study, AP was selected as the optimization performance metric because the datasets were imbalanced and AP provides a threshold independent measure of the classifier performance on predicting positive instances. The hyperparameter tuning was done with grid search over hyperparameter grids for LR, RF and XGB classifiers, with hyperparameter grids shown in Appendix B. The grid ranges covered both simple and more complex models while limiting model complexity to reduce the risk of overfitting. For the tree-based models (XGB and RF), the max_depth ranged from shallow (2–3) to moderately complex trees (8). Preliminary experiments indicated that expanding the search space beyond the chosen ranges did not yield notable performance improvements.

The optimal hyperparameters obtained from the grid search were following:

- LR_1_ : {penalty = “L2”, C = 0.2336}
- LR_2_ : {penalty = “L2”, C = 0.0886}
- RF_1_ : {n_estimators = 300, max_depth = 7, min_samples_split = 2, min_samples_leaf = 50}
- RF_2_ : {n_estimators = 400, max_depth = 6, min_samples_split = 15, min_samples_leaf = 5}
- XGB_1_ : {n_estimators = 400, max_depth = 4, learning_rate = 0.01, gamma = 2}
- XGB_2_ : {n_estimators = 150, max_depth = 3, learning_rate = 0.05, gamma = 2}
- XGB_baseline, 1_ : {n_estimators = 200, max_depth = 3, learning_rate = 0.03, gamma = 8}
- XGB_baseline, 2_ : {n_estimators = 300, max_depth = 3, learning_rate = 0.05, gamma = 0}

#### 2.3.4 Uncertainty estimation

To assess the statistical uncertainty of the estimated classifier performance, bootstrap confidence intervals were applied to the performance metrics. The confidence intervals provide an estimate of the variability arising from the finite size of the test set and enable a more robust comparison between classifier models. Considerably overlapping confidence intervals between classifiers were considered indicating of no clear performance differences between the models.

After model training and hyperparameter tuning, the final classifier was evaluated on the test set. Bootstrap samples were generated by taking random samples of the test set using sampling with replacement. The bootstrap samples preserved the same size as the test set. The analysis was performed independently for each classifier and dataset. A fixed random seed of 42 was used to ensure reproducibility.

For each bootstrap iteration, the performance metrics AP, precision, recall, BA, and F1-score were calculated. A total of 5,000 bootstrap iterations were performed. The distributions of the performance metrics obtained from the bootstrap samples were used for estimating the 95% confidence intervals of the metrics.

#### 2.3.5 Class imbalance handling

Algorithm dependent strategies were employed to account for the class imbalance in the dataset. For XGB, class imbalance was addressed using the scale_pos_weight, calculated as the ratio of negative to positive class samples in the training data. This increased the penalty associated with misclassifying positive instances during model training, which made the model focus more on the positive class in proportion with the weight parameter. For RF and LR, imbalance was handled through class weighting by setting class_weight=~balanced~, which automatically assigns class weights inversely proportional to class frequencies in the training set. GNB was trained using class frequency sample weights, where each training datapoint was weighted based on its class. All aforementioned methods increased the contribution of positive class samples during model training.

Hyperparameter tuning was performed using AP as the scoring metric, which is suitable for imbalanced classification problems because it emphasizes correct identification of the minority class. Furthermore, the final classification threshold for each model was determined by maximizing the F1-score, balancing the precision and recall metrics.

#### 2.3.6 Baseline models

To assess whether the proposed machine learning models provide predictive value beyond simple approaches or reduced set of features, baseline models were included in the evaluation.

The first baseline was a Null classifier, which predicts a positive class for all observations. This baseline corresponds to the expected performance obtained without any feature information. The Null classifier provided a lower reference level for the average precision (AP). The classifiers were denoted as Null_1_ for dataset 1 and Null_2_ for dataset 2.

The second baseline was a rule-based classifiers using a single environmental feature, denoted as RB_1_ for dataset 1 and RB_2_ for dataset 2. The classifier predicts a squeal event when the feature is within a specific interval. The lower and upper bounds of the interval were determined with a grid search using the training set. The grid consisted of 70 evenly spaced discrete values from the minimum value of the feature to the maximum value of the feature. For each candidate interval, predictions were made on the test set and the AP was calculated. The interval that resulted in highest AP was selected as the optimal rule for the classifier. Furthermore, the same grid search was done for all the environmental features (Features 1–11 in Table 1), and the single feature with the best AP was selected as the optimal feature for the classifier. The grid search resulted in following features and rules for the classifiers:

- RB_1_ : Positive class if μ_*clean*_ is within [0.352, 0.437], else negative class
- RB_2_ : Positive class if Δ*T*_*R*−*D*_ is within [5.037, 28.081], else negative class

This baseline provides a benchmark for assessing how much the machine learning models improve prediction performance compared to simple threshold-based decision rule of single feature.

The third baseline consisted of XGB classifiers trained only on directly measured variables (*T*_*air*_, *T*_*rail*_, *H*_*R, air*_, and time variables *h*_*sin*_ and *h*_*cos*_). These models were used to assess the added value of the derived variables, including temperature differences, derived humidity variables and estimated friction coefficients. The classifiers were denoted as XGB_1, measured_ for dataset 1 and XGB_2, measured_ for dataset 2.

#### 2.3.7 Feature importance analysis

To assess feature importances of the predictions, SHAP (SHapley Additive exPlanations) (Lundberg and Lee, 2017; Lundberg et al., 2020) analysis was used. SHAP evaluates the contribution of each feature on the model's prediction. For each prediction instance, a SHAP value is assigned for each feature stating how much the specific feature contributed to the overall prediction. This is done by comparing the outputs of all the possible feature subsets with and without the specific feature. The SHAP value indicates the importance of each feature on the prediction and whether the feature value affected the prediction toward positive or negative class. A positive SHAP value means that the feature contributed toward a positive class prediction, a negative SHAP value means that the feature contributed toward a negative class prediction, and a zero SHAP value means that the feature did not contribute to the prediction. To get an overall feature importance for a specific feature, the mean absolute value of all the SHAP values for the feature over all predictions can be calculated. Thus, SHAP analysis provides both explanations on specific prediction instances, and global explanations of the general feature importance over a dataset.

Additionally, the feature importances were calculated using the mean decrease in impurity (MDI) method for tree-based models. In this method, the decrease in gini impurity is calculated for each feature at each node, after which an average is calculated over all gini impurities. The MDI scores can then be used to rank the features in the order of importance, where a higher score means higher importance for the prediction.

Comparison of MDI rankings and SHAP rankings was used to assess the consistency and robustness of feature importance estimates. While SHAP values where used as primary estimated of feature importance, MDI values were used to assess the consistency and robustness of the estimates.

Regarding the features for hour of day, the individual feature importances of the two components *h*_*sin*_ and *h*_*cos*_ were summed together into a combined feature importance for a single feature called “Hour of day.” This was done for better interpretability of the feature importance since the two components describe the same variable. The procedure was applied for both SHAP and MDI analysis.

### 2.4 Operational assessment for predictive TOR-FM application

To assess the practical usefulness of the developed prediction models, an operational evaluation was conducted using a hypothetical top-of-rail friction modifier (TOR-FM) control strategy for a wayside system to mitigate squealing. A common standard practice is to apply TOR-FM for every train pass-by, corresponding to continuous application and maximum product consumption. The prediction models were evaluated as a decision-support tool for triggering TOR-FM application.

For each prediction threshold, train pass-bys predicted as positive were assumed to trigger TOR-FM application, while pass-bys predicted as negative were assumed to not trigger the application. The percentage of train pass-bys receiving TOR-FM was defined as the trigger rate. TOR-FM product savings were calculated relative to the standard practice of applying the product for all train pass-bys.

The effectiveness of the predictive strategy was quantified using recall, representing the proportion of actual squeal events correctly identified by the model. In this context, recall corresponds to the percentage of squeal events that trigger TOR-FM application. Operational trade-off was assessed by analyzing the relationship between trigger rate and recall for a range of decision thresholds. The analysis was performed on the test set and is intended as a proof-of-concept assessment of how squeal prediction using environmental data could support adaptive TOR-FM control.

## 3 Results

The following subsections cover the results in terms of prediction performance and feature importance assessment. Subsection 3.1 presents the general prediction performance of the classifiers. Subsection 3.2 shows the performance comparison with baseline models. Subsection 3.3 shows the feature importance analysis and Subsection 3.4 shows the results from operational assessment of TOR-FM triggering.

### 3.1 Prediction performance

**Figure 12:**
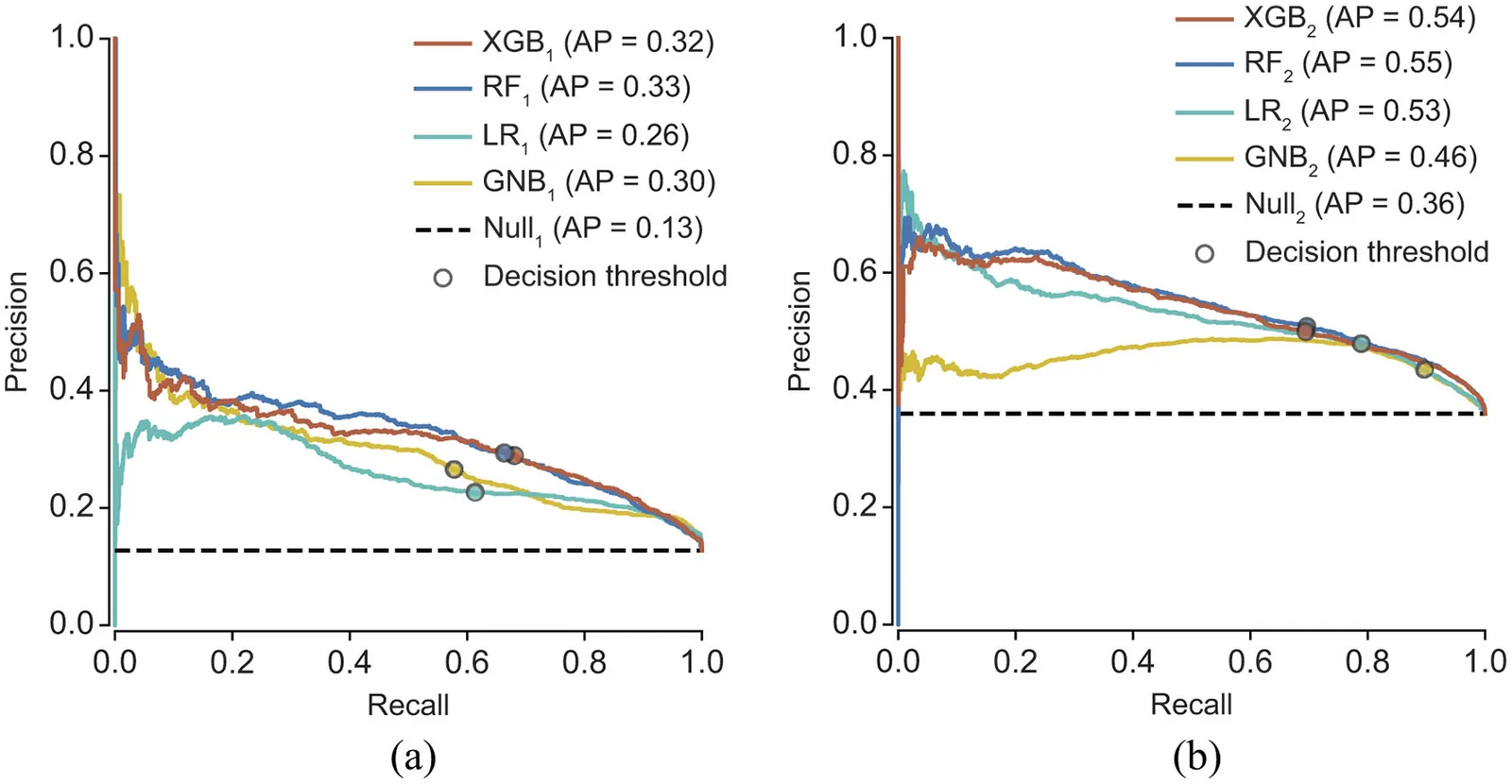
Precision-recall curve (PR-curve), average precision (AP) and decision threshold of each classifier. **(a)** Dataset 1: squealing on the low-rail, and **(b)** Dataset 2: squealing on the high-rail.

The prediction performance was assessed by inspecting the test set performance scores and PR-curves of the classifiers. These scores include AP and the confusion matrix based metrics precision, recall, BA, and F1-score. PR-curves and AP scores, along with decision thresholds, are shown in Figure 12a for dataset 1 and Figure 12b for dataset 2. All scores, decision thresholds and 95% bootstrap confidence intervals are summerized in Table 3 for dataset 1 and Table 4 for dataset 2. The scores of Null classifier denote the baseline performance when all instances are predicted as positive class.

**Table 3:** Performance scores and optimized decision thresholds of classifiers trained on dataset 1.

Metric	XGB_1_	RF_1_	LR_1_	GNB_1_	Null_1_
Precision	0.29 [0.27–0.31]	0.29 [0.28–0.31]	0.23 [0.21–0.24]	0.27 [0.25–0.28]	0.13 [0.12–0.13]
Recall	0.68 [0.65–0.70]	0.66 [0.64–0.69]	0.61 [0.59–0.64]	0.58 [0.55–0.61]	1.00
BA	0.72 [0.71–0.73]	0.72 [0.70–0.73]	0.65 [0.64–0.67]	0.67 [0.66–0.69]	0.50
AP	0.32 [0.30–0.34]	0.33 [0.30–0.35]	0.26 [0.24–0.28]	0.30 [0.27–0.32]	0.13 [0.12–0.13]
F1-score	0.41 [0.39–0.42]	0.41 [0.39–0.43]	0.33 [0.31–0.35]	0.36 [0.35–0.38]	0.23 [0.22–0.24]
Threshold	0.64	0.63	0.62	0.98	-

Bootstrap 95% confidence intervals are shown in brackets.

**Table 4:** Performance scores and optimized decision thresholds of classifiers trained on dataset 2.

Metric	XGB_2_	RF_2_	LR_2_	GNB_2_	Null_2_
Precision	0.50 [0.49–0.51]	0.51 [0.49–0.52]	0.48 [0.47–0.49]	0.44 [0.42–0.45]	0.36 [0.35–0.37]
Recall	0.70 [0.68–0.71]	0.70 [0.68–0.71]	0.79 [0.78–0.80]	0.90 [0.89–0.91]	1.00
BA	0.65 [0.64–0.66]	0.66 [0.65–0.67]	0.65 [0.65–0.66]	0.62 [0.61–0.63]	0.50
AP	0.54 [0.53–0.56]	0.55 [0.53–0.57]	0.53 [0.51–0.55]	0.46 [0.44–0.47]	0.36 [0.35–0.37]
F1-score	0.58 [0.57–0.59]	0.59 [0.58–0.60]	0.60 [0.58–0.61]	0.59 [0.58–0.60]	0.53 [0.52–0.54]
Threshold	0.53	0.54	0.44	0.10	-

Bootstrap 95% confidence intervals are shown in brackets.

It should be noted that all metrics, except for AP, are dependent on the chosen decision thresholds that maximize the F1-scores. These thresholds can be adjusted after the prediction to gain a wanted balance between precision and recall. As an example, for the case of predicting squeal events for TOR-FM lubrication triggering, a threshold that results in high recall might be desirable in order to predict a high number of correct squeal events. Thus, the decision threshold could be moved rightwards along the PR-curve to increase the recall. This would decrease the precision, but for the case of TOR-FM triggering, false positives in the prediction are not crucial.

Among the chosen metrics, AP and PR-curves are considered the most appropriate for the overall evaluation and comparison of classifier performances. Unlike threshold dependent metrics, these summarize performance across all decision thresholds, providing a measure of the model's general predictive capability.

For both datasets, all machine learning classifiers outperformed the Null classifier. The tree-based models, XGB and RF, achieved the highest predictive performance, whereas LR and GNB generally exhibited lower scores.

For dataset 1, XGB and RF showed nearly identical performance, achieving AP values of 0.32 and 0.33, respectively, and identical F1-scores of 0.41. Their confidence intervals overlapped notably, indicating of no clear difference in performance between the models. Both tree-based models also achieved the highest balanced accuracy of 0.72. LR and GNB produced lower AP values of 0.26 and 0.30, respectively. Although GNB shows a relatively high AP value, the tree-based models show clearly higher precision values in the PR-curve for recall values from 0.5 to 0.9, when compared to both LR and GNB. While all classifiers outperformed the Null classifier, the overall predictive performance was moderate, highlighting the complexity of the prediction of low-rail squeal.

For dataset 2, all classifiers achieved higher predictive performance than for dataset 1, however, the relative improvement in performance when compared to Null classifier was similar. XGB and RF achieved nearly identical AP of 0.54 and 0.55, respectively. Similarly, the F1-scores were nearly identical (0.59 for RF and 0.58 for XGB), with significant overlap in confidence intervals. LR obtained almost the same performance as the tree-based models, whereas GNB exhibited lower scores. Notably, all the classifiers showed similar performance for recall values over 0.7 in the PR-curves.

### 3.2 Comparison with baseline performance

Comparison with baseline models provide insight into the predictive value of increasing feature set and complexity. The Null classifier represents the performance without using any feature data, while the RB classifier evaluates whether a simple threshold on a single environmental feature is sufficient for squeal prediction. The XGB_measured_ model demonstrates the predictive value of using directly measured environmental features alone. Comparison with the full XGB model therefore quantifies the additional predictive value gained by using the additional engineered features.

**Figure 13:**
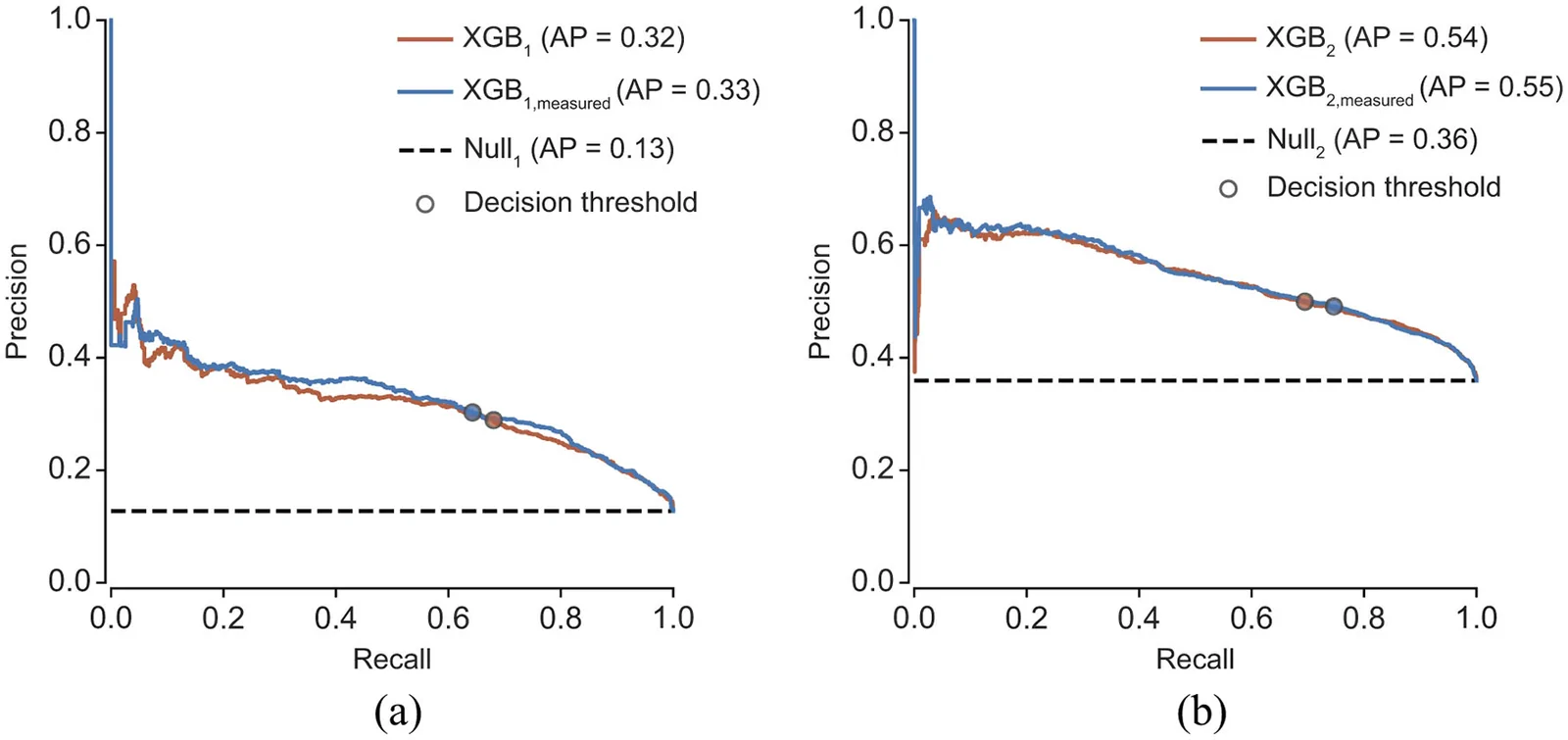
Precision-recall curve (PR-curve), average precision (AP) and decision threshold of XGB and XGB_measured_ classifiers. **(a)** Dataset 1: squealing on the low-rail, and **(b)** Dataset 2: squealing on the high-rail.

Tables 5, 6 compare the performance of the full XGB model with the baseline models for dataset 1 and 2, respectively. Figures 13a, b show the PR-curves of XGB and XGB_measured_ classifiers.

**Table 5:** Performance scores and optimized decision thresholds of classifiers trained on dataset 1.

Metric	XGB_1_	XGB_1, measured_	RB_1_	Null_1_
Precision	0.29 [0.27–0.31]	0.30 [0.30–0.35]	0.20 [0.19–0.22]	0.13 [0.12–0.13]
Recall	0.68 [0.65–0.70]	0.64 [0.62–0.67]	0.69 [0.67–0.72]	1.00
BA	0.72 [0.71–0.73]	0.71 [0.70–0.73]	0.65 [0.63–0.66]	0.50
AP	0.32 [0.30–0.34]	0.33 [0.30–0.35]	-	0.13 [0.12–0.13]
F1-score	0.41 [0.39–0.42]	0.41 [0.39–0.43]	0.31 [0.30–0.33]	0.23 [0.22–0.24]
Threshold	0.64	0.64	-	-

Bootstrap 95% confidence intervals are shown in brackets.

**Table 6:** Performance scores and optimized decision thresholds of classifiers trained on dataset 2.

Metric	XGB_2_	XGB_2, measured_	RB_2_	Null_2_
Precision	0.50 [0.49–0.51]	0.49 [0.48–0.50]	0.49 [0.48–0.50]	0.36 [0.35–0.37]
Recall	0.70 [0.68–0.71]	0.75 [0.73–0.76]	0.69 [0.67–0.70]	1.00
BA	0.65 [0.64–0.66]	0.67 [0.65–0.67]	0.64 [0.63–0.65]	0.50
AP	0.54 [0.53–0.56]	0.55 [0.53–0.56]	-	0.36 [0.35–0.37]
F1-score	0.58 [0.57–0.59]	0.59 [0.58–0.60]	0.57 [0.56–0.58]	0.53 [0.52–0.54]
Threshold	0.53	0.52	-	-

Bootstrap 95% confidence intervals are shown in brackets.

For dataset 1, the XGB_1, measured_ trained only on the measured environmental variables achieved performance comparable to the full XGB_1_ model. The AP and BA differed only by 0.01, while the F1-score was the same and the confidence intervals showed a significant overlap. Also, the PR-curves between the classifiers are almost identical. These results indicate that the directly measured environmental variables contain most of the predictive information. The RB_1_ classifier achieved lower performance than the tree-based models, with an F1-score of 0.31 and balanced accuracy of 0.65. This demonstrates that using multiple environmental variables and a machine learning approach provides better predictive performance than a simple decision rule on a single feature. AP was not calculated for the RB classifiers, as the classifier prediction is deterministic and not considered in probabilities where thresholds could be applied.

For dataset 2, the XGB_2, measured_ again performed similarly to the full XGB_2_ model. The AP and F1-score differed by 0.01, while the BA differed by 0.02. Also, the PR-curves between the classifiers are almost identical. Notably, the RB_2_ classifier achieved almost similar performance (F1-score = 0.57, BA = 0.64) as the tree-based models. This indicates that the single environmental variable contains substantial predictive information for high-rail squeal prediction.

Overall, the baseline comparison indicates that directly measured environmental features alone contain most of the predictive information regarding curve squeal occurrence. The use of multiple environmental variables within a machine learning model outperformed a single-feature threshold rule for dataset 1, while for dataset 2 the single feature threshold rule showed a performance similar to tree-based models. However, the single feature threshold models do not produce probability outputs and operate at a single fixed decision point. Thus, application specific threshold tuning of the precision-recall trade-off is not possible.

### 3.3 Feature importances

Since XGB and RF models achieved comparable predictive performance, feature importance was evaluated for both tree-based models using SHAP and MDI analysis. Although the absolute SHAP and MDI values differ between models, the feature rankings enable comparing the relative importance of the environmental variables.

The two algorithms identified similar dominant predictors for both datasets, indicating that the feature rankings are robust with respect to the choice of tree-based model. Consequently, the detailed feature dependent SHAP dependence analysis is presented only for XGB to illustrate how the most influential predictors affect the model output. The feature importances for dataset 1 are shown in Subsection 3.3.1 and for dataset 2 in Subsection 3.3.2.

#### 3.3.1 Dataset 1—Squealing on low-rail

**Figure 14:**
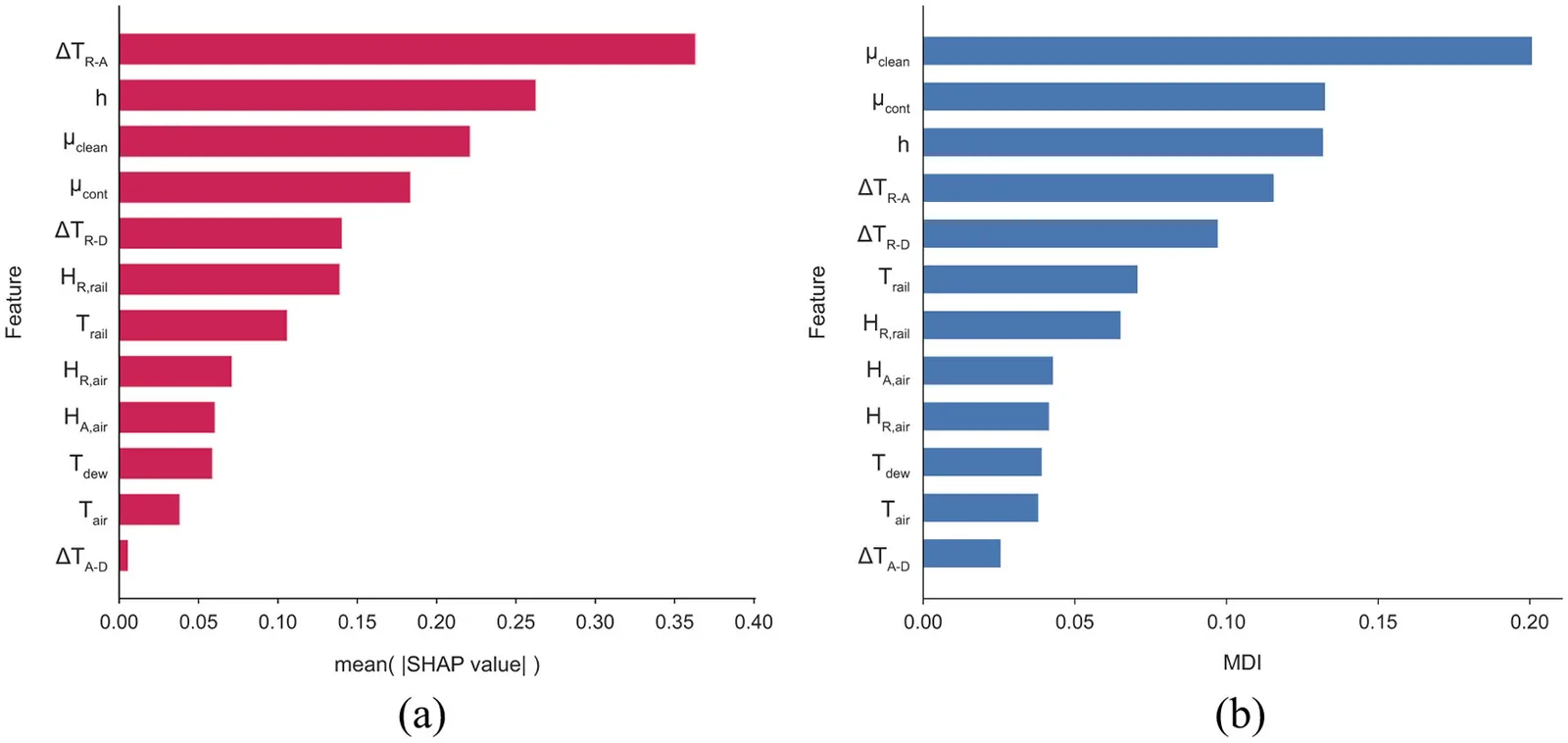
Feature ranking of the XGB_1_ classifier. **(a)** SHAP analysis and **(b)** MDI analysis.

Figure 14 shows the mean absolute SHAP values and MDI values for features of the XGB_1_ classifier ranked in the order of importance. Both SHAP and MDI identified a subset of variables as the dominant contributors to the model predictions. Although the exact ranking differed between SHAP and MDI, an agreement regarding the most important features was seen. SHAP assigned the highest feature importance for rail–air temperature difference (Δ*T*_*R*−*A*_), followed by hour of the day (*h*), the estimated friction coefficients (μ_*clean*_ and μ_*cont*_), and the rail–dew point temperature difference (Δ*T*_*R*−*D*_). MDI assigned the same features as being the most important but in a different order, with friction coefficients having the highest importance.

**Figure 15:**
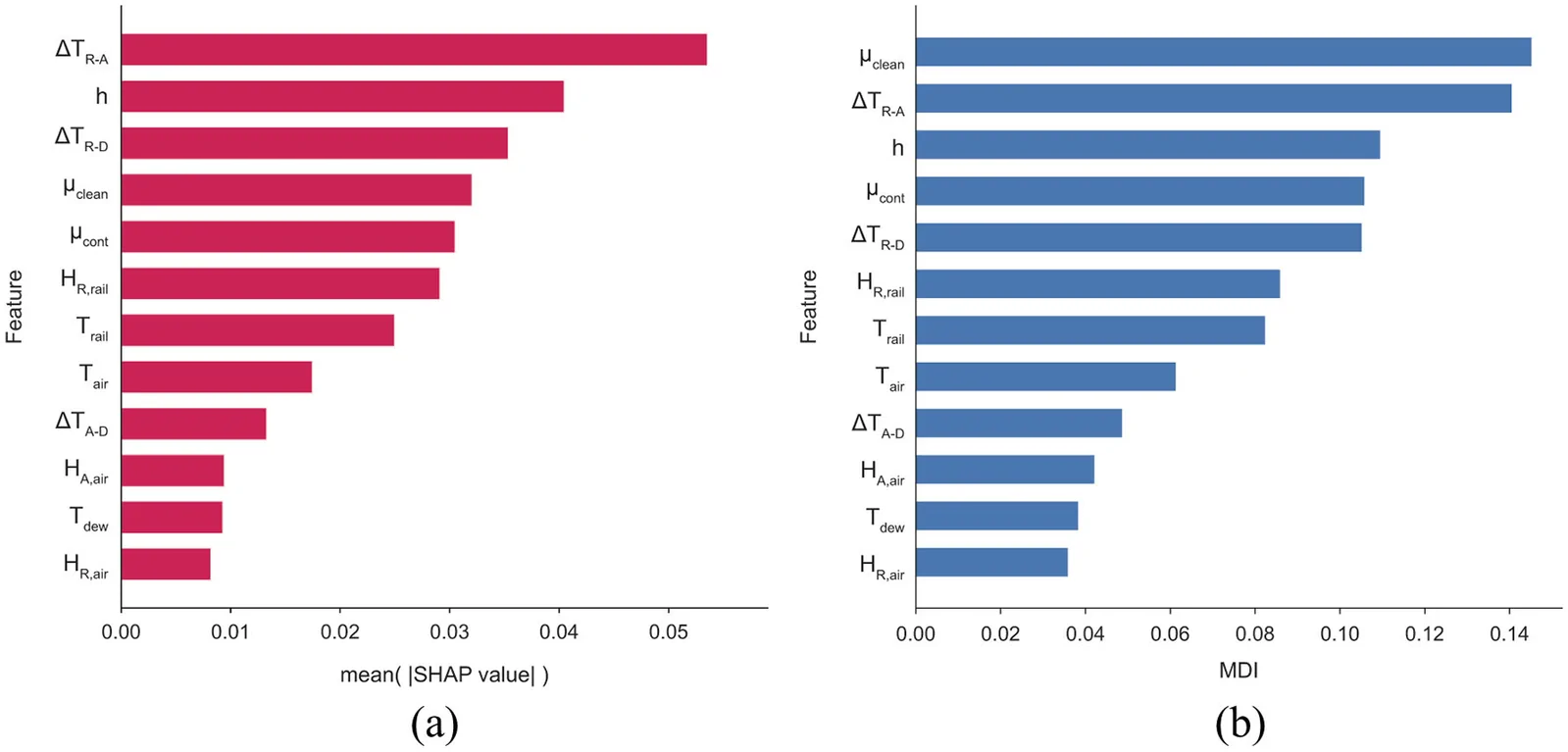
Feature ranking of the RF_1_ classifier. **(a)** SHAP analysis and **(b)** MDI analysis.

Figure 15 shows the same feature ranking of SHAP and MDI importances for the RF_1_ classifier. The RF model identified a similar group of strong predictors. Both SHAP and MDI assigned the highest importance to rail–air temperature difference (Δ*T*_*R*−*A*_), hour of the day (*h*), the estimated friction coefficients (μ_*clean*_ and μ_*cont*_), and the rail–dew point temperature difference (Δ*T*_*R*−*D*_). Minor differences were observed in the order of these features, but the overall agreement between XGB and RF indicates that the identified environmental predictors are robust with respect to the selected tree-based algorithm.

**Figure 16:**
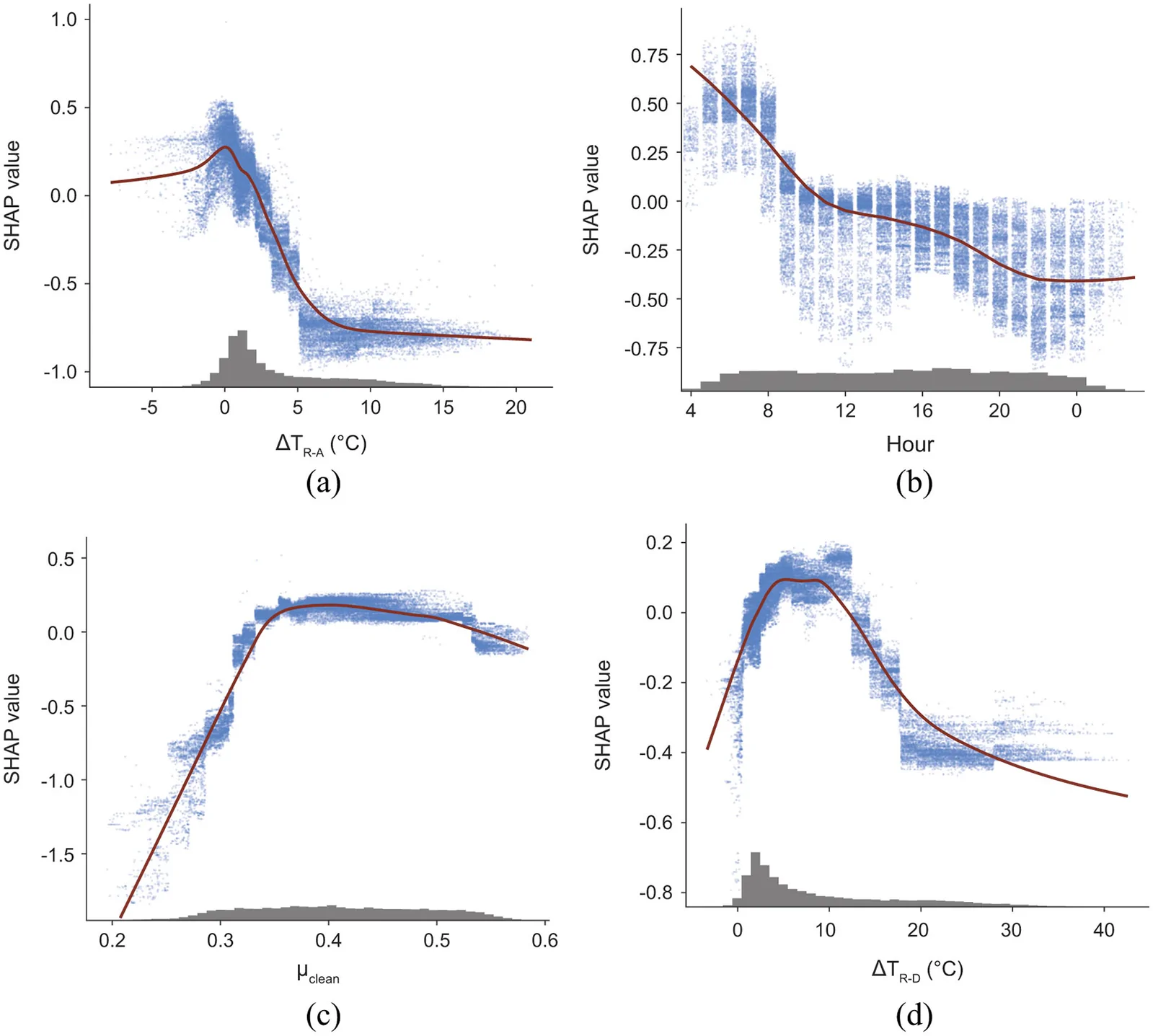
SHAP values of four influential features of XGB_1_ classifier: **(a)** difference between the rail temperature and air temperature Δ*T*_*R*−*A*_, **(b)** hour of day *h*, **(c)** estimated friction for clean contact μ_*clean*_ and **(d)** difference between the rail temperature and dew point Δ*T*_*R*−*D*_. All individual SHAP values are displayed on a scatter plot. A regression line based on the scatter plot is presented with a solid red line. The histogram of each feature is presented in the lower part of each graph.

Figure 16 shows the individual SHAP values as scatter plots and corresponding regression lines for four influential features of XGB_1_, namely Δ*T*_*R*−*A*_, *h*, μ_*clean*_ and Δ*T*_*R*−*D*_. The scatter plots consist of all the individual feature values and their corresponding SHAP values. The solid line shows the regression line based on scatter plots using locally estimated scatter plot smoothing regression (LOESS) (Jacoby, 2000). Bars in the bottom of each graph show the histogram of the feature values.

The SHAP values reveal non-linear relationships, indicating that the model identifies specific feature ranges associated with increased or decreased estimated risk for squeal. For Δ*T*_*R*−*A*_ the highest positive SHAP values are seen at small feature values. Above approximately 3 °C, the SHAP values are consistently negative. Hour of the day (*h*) shows positive SHAP values during morning hours, and decreasing negative SHAP values throughout the day. This trend is consistent with the previously observed daily variation in squeal occurrence and likely reflects the influence of changing environmental conditions throughout the day. For μ_*clean*_ the SHAP values are negative for low feature values, and increase rapidly as the feature values approach approximately 0.35, after which the SHAP values remain elevated and slightly above zero until a small trend toward negative SHAP values is seen for feature values above approximately 0.53. The difference between rail temperature and dew point (Δ*T*_*R*−*D*_) reveals a range at approximately 5–10 °C where SHAP values are positive. Outside of this range the SHAP values are negative.

#### 3.3.2 Dataset 2—Squealing on high-rail

**Figure 17:**
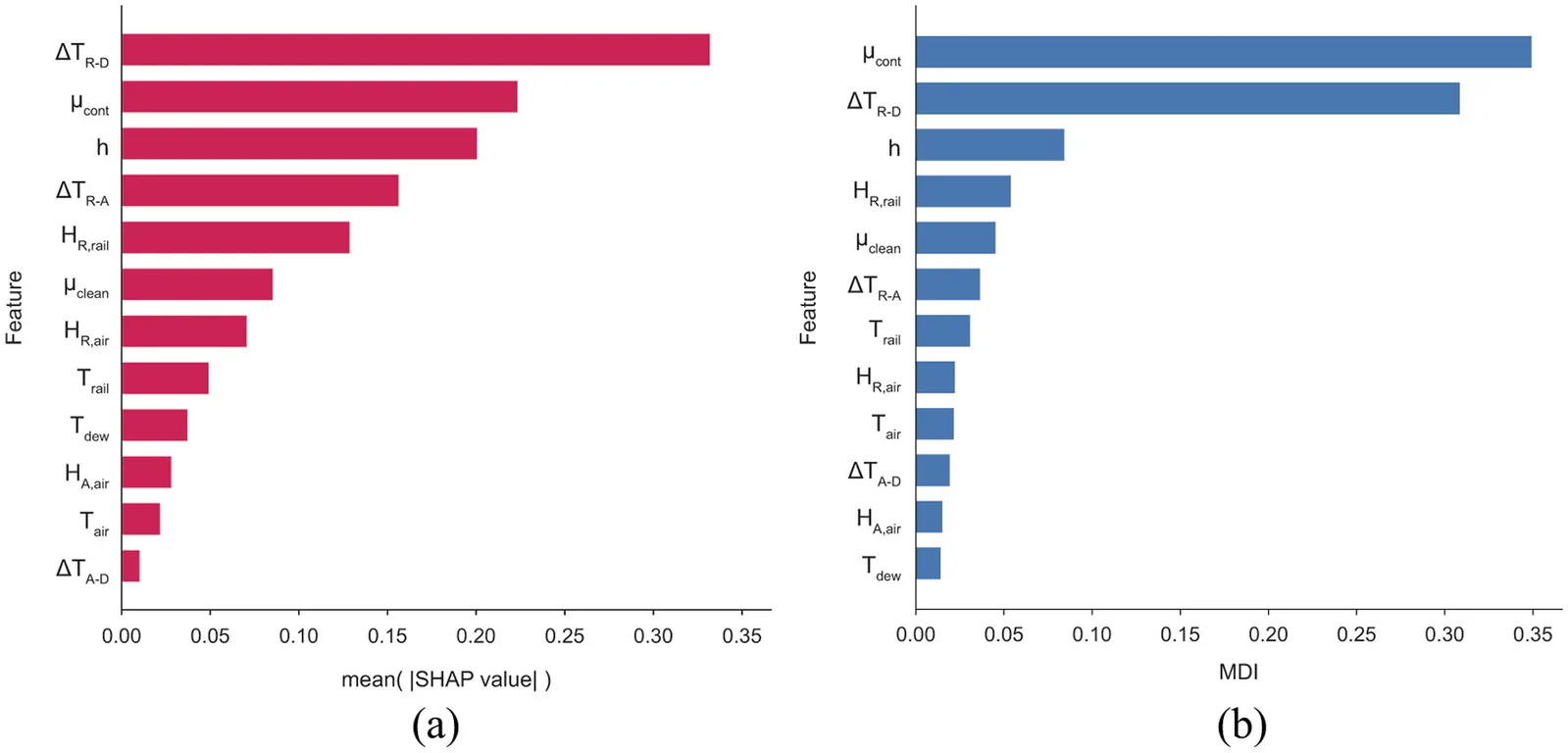
Feature ranking of the XGB_2_ classifier. **(a)** SHAP analysis and **(b)** MDI analysis.

Figure 17 shows the mean absolute SHAP values and MDI values for the features of XGB_2_ classifier ranked in the order of importance. The ranking shows that the rail–dew point temperature difference (Δ*T*_*R*−*D*_) and the estimated contaminated friction coefficient (μ_*cont*_) are dominating features, followed by hour of the day (*h*). In MDI ranking the dominance of the two highest ranking features is especially evident, while SHAP ranking shows a slightly more even ranking.

**Figure 18:**
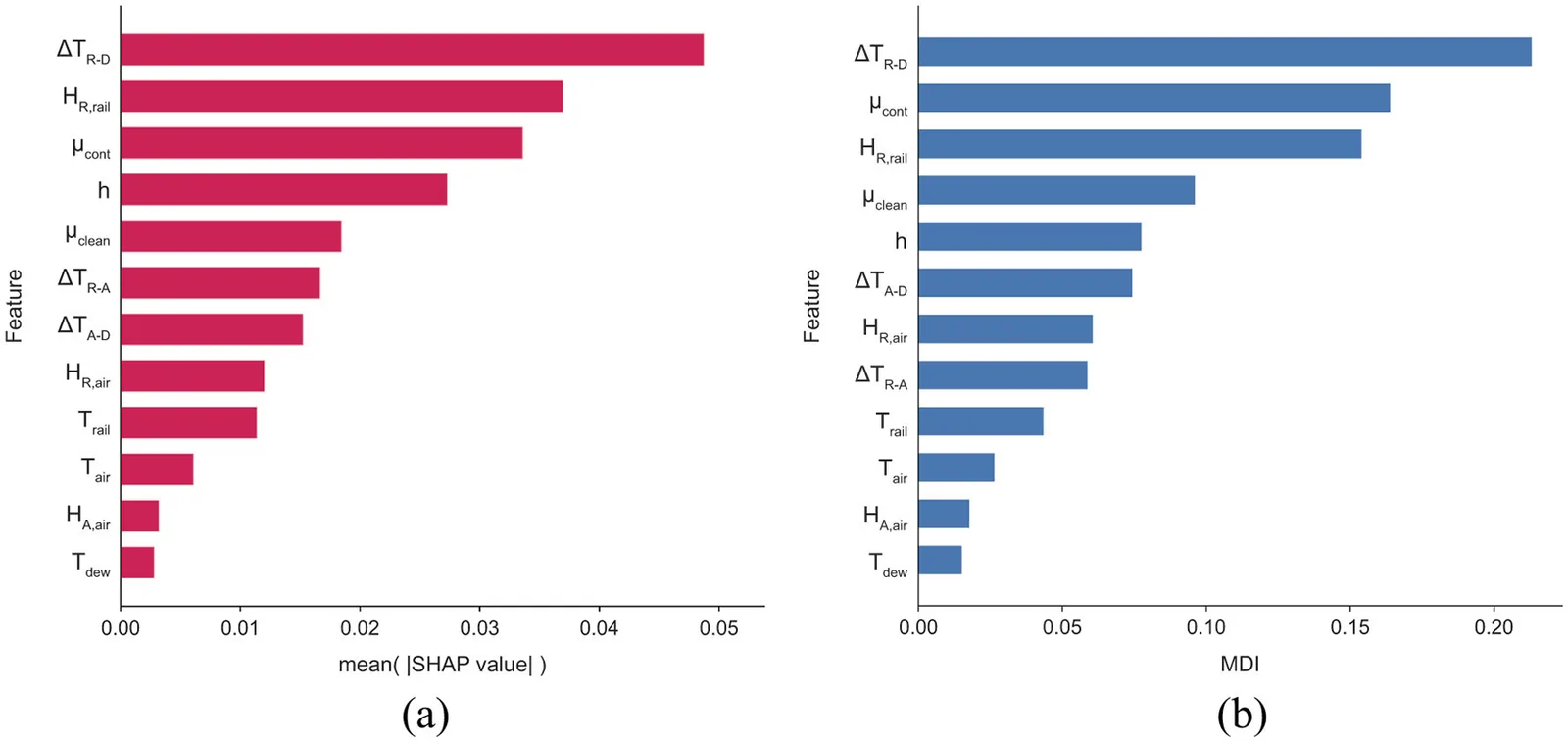
Feature ranking of the RF_2_ classifier. **(a)** SHAP analysis and **(b)** MDI analysis.

Figure 18 shows the feature ranking of SHAP and MDI importances for the RF_2_ classifier. Feature rankings of RF_2_ were consistent with those obtained from XGB_2_. The rail–dew point temperature difference (Δ*T*_*R*−*D*_), estimated contaminated friction coefficient (μ_*cont*_), hour of the day (*h*), and relative rail humidity (*H*_*R, rail*_) remained among the most important predictors. Although the relative ordering differed slightly, the agreement between the two algorithms supports the robustness of the identified feature importance patterns.

**Figure 19:**
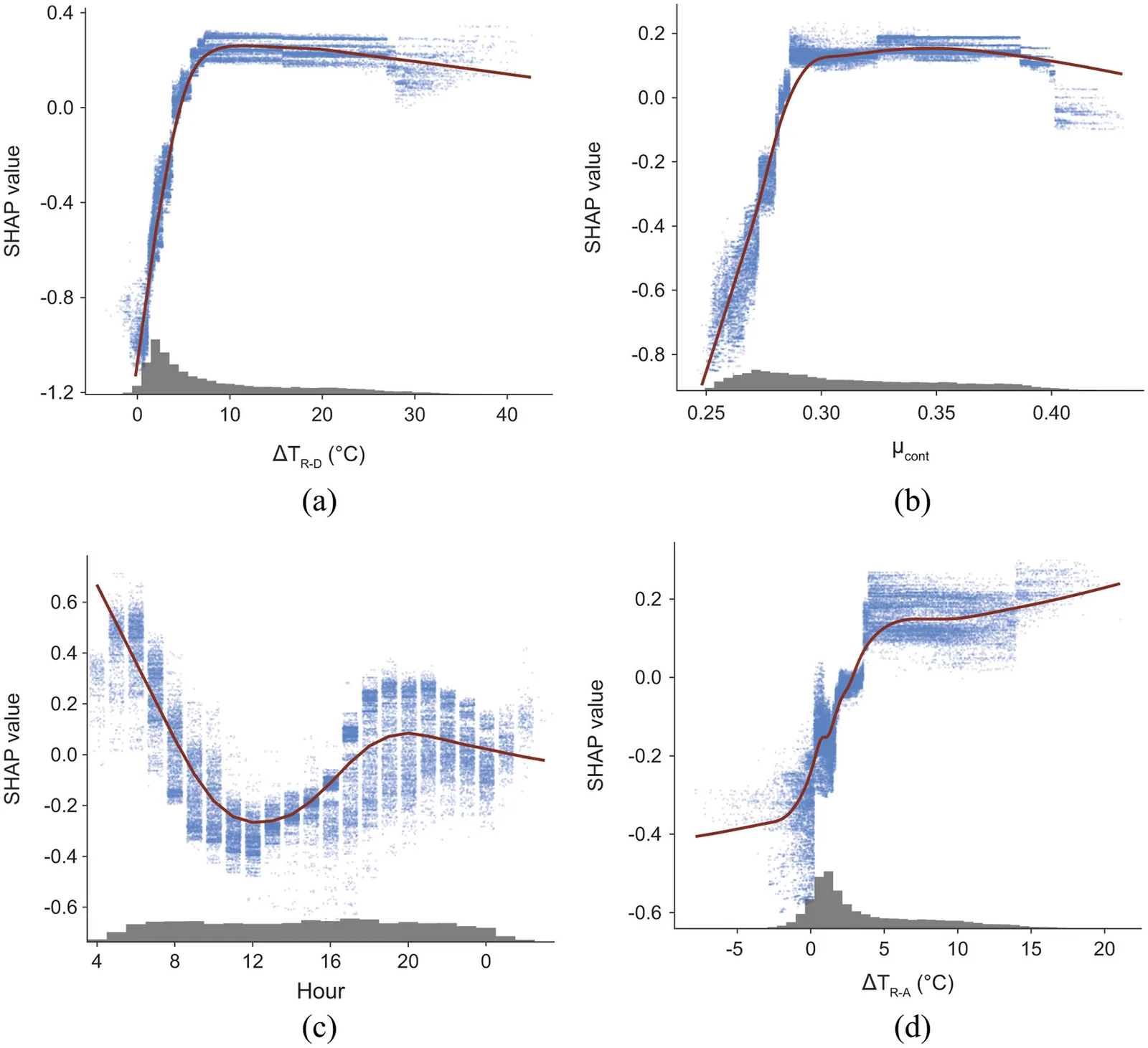
SHAP values of the four notably influential features of XGB_2_ classifier: **(a)** difference between the rail temperature and dew point Δ*T*_*R*−*D*_, **(b)** estimated friction for contaminated contact μ_*cont*_, **(c)** hour of day *h* and **(d)** difference between the rail temperature and air temperature Δ*T*_*R*−*A*_. All individual SHAP values are displayed on a scatter plot. A regression line based on the scatter plot is presented with a solid red line. The histogram of each feature is presented in the lower part of each graph.

Figure 19 shows the SHAP values for four influential features of the XGB_2_ classifier, namely Δ*T*_*R*−*D*_, μ_*cont*_, *h* and Δ*T*_*R*−*A*_. The scatter plot consists of all the individual feature values and their corresponding SHAP values. The solid line in the plots show the LOESS regression line based on the scatter plots. The bars in the bottom of each graph shows the histogram of the feature values over the dataset.

For Δ*T*_*R*−*D*_ the small feature values close to zero are assigned strong negative SHAP values. The SHAP values increase rapidly with feature values until for feature values above approximately 5 °C the SHAP values remain constant and positive. The estimated friction coefficient μ_*cont*_ shows a similar trend, with low friction values assigned with negative SHAP values and feature values above 0.28 assigned with mostly positive SHAP values. Hour of day (*h*) show two regions with mostly positive SHAP values, namely morning hours and evening hours, with negative SHAP values during midday. For Δ*T*_*R*−*A*_ the SHAP values increase nearly monotonically, with a clear step from negative to positive SHAP values at 0–5 °C.

### 3.4 Operational assessment for TOR-FM systems

**Figure 20:**
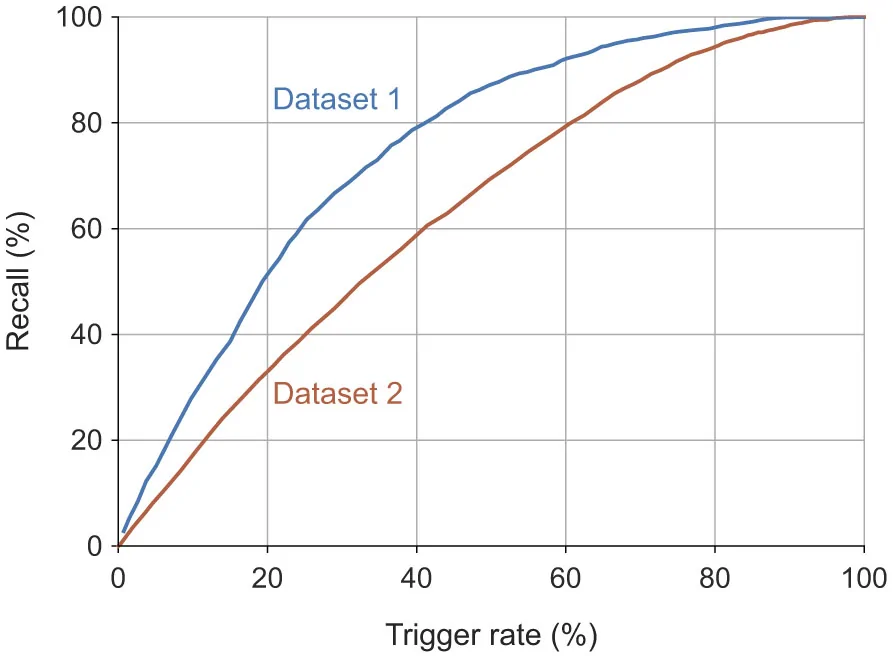
Tradeoff curve between TOR-FM trigger rate and recall for XGB_1_ on dataset 1 and XGB_2_ on dataset 2.

Figure 20 demonstrates the trade-off between TOR-FM trigger rate and recall (rate of correctly detected squeal events) for the XGB classifiers on dataset 1 and 2. Tables 7, 8 present example operating points corresponding to different target recall levels for the same classifiers.

**Table 7:** Target recall and corresponding decision threshold, recall, precision, trigger rate and savings in comparison to 100% trigger rate for XGB_1_ on dataset 1.

Target recall	Decision threshold	Recall	Precision	Trigger rate (%)	Savings (%)
0.95	0.34	0.950	0.182	66.6	33.4
0.90	0.46	0.901	0.205	55.8	44.2
0.85	0.53	0.856	0.231	47.2	52.8
0.80	0.56	0.813	0.242	42.7	57.3

**Table 8:** Target recall and corresponding decision threshold, recall, precision, trigger rate and savings in comparison to 100% trigger rate for XGB_2_ on dataset 2.

Target recall	Decision threshold	Recall	Precision	Trigger rate (%)	Savings (%)
0.95	0.31	0.952	0.421	81.3	18.7
0.90	0.40	0.906	0.442	73.7	26.3
0.85	0.46	0.856	0.462	66.6	33.4
0.80	0.48	0.815	0.469	62.5	37.5

For low-rail squeal prediction (XGB_1_), notable reductions in trigger rate were achieved while maintaining a high recall. At a target recall of 90%, the model correctly identified 90.1% of squeal events while triggering TOR-FM for only 55.8% of train pass-bys, corresponding to a 44.2% reduction in consumption compared with continuous TOR-FM application. At a target recall of 85%, TOR-FM trigger rate was reduced by 52.8% while still capturing 85.6% of squeal events.

For high-rail squeal prediction (XGB_2_), the achievable reductions were smaller but still notable. At a target recall of 90%, trigger rate was reduced by 26.3%, while 90.6% of squeal events were triggered with TOR-FM. At a target recall of 85%, the reduction increased to 33.4%.

These results suggest that environmental data based prediction may enable more targeted TOR-FM application strategies compared with continuous application. In particular, the low-rail squeal model demonstrated the ability to identify periods with low squeal risk, with potential for notably reducing TOR-FM consumption while maintaining a high detection rate of squeal events. The optimal operating point depends on the relative importance of lubricant consumption, maintenance effort, and missed squeal events.

## 4 Discussion

### 4.1 Predictive capability

All evaluated machine learning models outperformed the Null classifier, with the tree-based models generally achieving the highest performance, suggesting that non-linear relationships between environmental variables contribute to the prediction. Interestingly, the Logistic Regression (LR) achieved predictive performance comparable to the tree-based models for high-rail squeal, whereas its performance was clearly lower for low-rail squeal. This suggests that the relationship between the environmental features and high-rail squeal may be more linear than for low-rail squeal. The comparable performance of XGB and RF indicates that the predictive information of the environmental variables is more important than the specific choice of algorithm.

The baseline models utilizing only measured variables (XGB_measured_) achieved predictive performance comparable to the full models. This suggests that directly measured environmental variables capture most of the predictive information available in the present dataset. The engineered variables appear to be alternative representations of directly measured features with little or no new predictive information. Specifically, the tree-based models seem to be able to learn the interrelationships of the measured features without needing to derive additional features based on these.

The limited predictive performance of the models is consistent with the fact that curve squeal is a complex phenomenon. Squeal arises from a combination of interacting mechanisms, including wheel–rail contact mechanics, frictional behavior, and vehicle–track dynamics. While environmental conditions affect frictional properties and thus contribute to squeal tendency, they represent only one part of the system. Consequently, it is reasonable that models based solely on environmental variables provide only limited predictive capability. Additional variables describing wheel–rail interaction, vehicle dynamics, operational parameters and surface condition are expected to improve predictive performance, but they require specialized instrumentation and measurements, and are difficult to obtain continuously during railway operation. The aim of this study was to investigate if environmental variables that are easily measurable on the wayside of the track can give predictive signals of squeal occurrence. Bearing this in mind, the results indicate that environmental variables have predictive value although the observed performance is limited.

### 4.2 Differences between low-rail and high-rail squeal prediction

The study shows that low-rail and high-rail squeal exhibit different predictive characteristics. The feature importance rankings and SHAP analyses show different results for the two rails. Although both models (XGB_1_ for low-rail and XGB_2_ for high-rail) identified temperature-related variables among the most important features, the learned relationships are different. The rail–dew point temperature difference Δ*T*_*R*−*D*_ and rail–air temperature difference Δ*T*_*R*−*A*_ showed nearly opposite SHAP trends for the two models. For these, the high feature values generally showed negative SHAP values for the low-rail model and positive SHAP values for the high-rail model.

The SHAP analysis also indicates that the daily pattern differs between the models. For low-rail squeal, the model assigned the highest predicted risk for squeal during the early morning, with a decrease throughout the day. In contrast, the high-rail model exhibited two periods of higher predicted risk, during the morning and evening hours, with a lower predicted risk around midday.

These observations are consistent with the statistical analysis presented in Toratti et al. (2026), which reported different environmental tendencies for low-rail and high-rail squeal occurrence. The differences support the hypothesis that low-rail and high-rail squeal are associated with different environmental conditions and possibly different mechanisms. This study demonstrates that these differences are also seen in predictive machine learning models and their feature importance patterns. Although the SHAP analysis does not serve as proof of causal mechanisms, the results indicate that modeling the two squeal types separately provides additional value when compared to treating curve squeal as a single phenomenon.

### 4.3 Physical interpretation of the features

The observed feature importances suggest that squeal occurrence is associated with different environmental conditions. The close agreement between the XGB and RF feature rankings suggests that the observed feature importance is robust and not specific to a particular tree-based algorithm. However, these results should be interpreted as model associations rather than evidence of physical mechanisms.

The temperature differences Δ*T*_*R*−*A*_ and Δ*T*_*R*−*D*_ were identified as important features for both low-rail and high-rail squeal prediction. The rail–air temperature difference Δ*T*_*R*−*A*_ provides information about the rail temperature relative to the surrounding air. Large values are generally associated with increased solar heating of the rail and enhanced water evaporation from the rail surface, whereas small values occur when the rail and surrounding air temperature are close to equal. Thus, Δ*T*_*R*−*A*_ may indicate a drying rail surface which influences the wheel–rail contact conditions.

The rail–dew point temperature difference Δ*T*_*R*−*D*_ provides an indication of the proximity of the rail temperature to the water vapor saturation point of surrounding air. Small values of Δ*T*_*R*−*D*_ indicate that the rail temperature approaches the dew point, increasing the likelihood of moisture formation or condensation on the rail surface. Such conditions are assumed to alter the wheel–rail friction characteristics, which are known to affect curve squeal generation.

The estimated friction coefficients and hour of day were also identified as important features by the models. The friction coefficients are not measurements of wheel–rail friction but are calculated from empirical relationships based on environmental variables. Consequently, they should be interpreted as indicators of environmental conditions associated with changes in friction rather than direct measurements. Their importance in the SHAP analysis suggests that the friction estimates provide useful predictive information. Future studies incorporating direct friction measurements would be required to verify these relationships.

The hour of day relates to the environmental variations that follow a daily cycle. These include changes in sun radiation, rail heating and cooling, evaporation, dew formation, and likely other conditions that are not represented by the measured environmental variables. The weak correlation observed between hour of day and the remaining environmental features suggests that it contains additional predictive information beyond the listed environmental variables.

The feature importances should also be considered in the context of the observed correlations between features. The correlation analysis showed that several features are strongly correlated. Consequently, the importance of individual features should not be interpreted as proof that they independently govern the predictions. Instead, the models appear to use multiple features that represent similar environmental conditions, with the predictive information distributed among them. This is further supported by the baseline analysis, which showed that models trained using only directly measured environmental variables achieved performance comparable to the full feature set. Thus, the feature rankings should be interpreted as indicating which environmental conditions are informative for prediction.

### 4.4 Operational assessment and practical implementation

The operational assessment indicates that the prediction models may provide value even though the classification performance is moderate. For predictive TOR-FM control, the objective is not necessarily to predict every squeal event with high precision, but rather to reduce unnecessary product consumption while maintaining detection of a large proportion of squeal events. For this case, false positives lead to additional TOR-FM consumption, whereas false negatives correspond to missed opportunities for squeal mitigation. Therefore, recall is of greater practical importance than precision.

The results demonstrate that notable reductions in TOR-FM trigger rate can be achieved while maintaining a high recall. This suggests that environmental data based prediction could be used as a decision-support tool for adaptive TOR-FM systems. However, the present analysis should be regarded as a proof-of-concept assessment, as the analysis does not account for the actual effectiveness of TOR-FM application, TOR-FM build-up from consecutive train pass-bys, or the costs related to TOR-FM system maintenance and product consumption.

**Figure 21:**
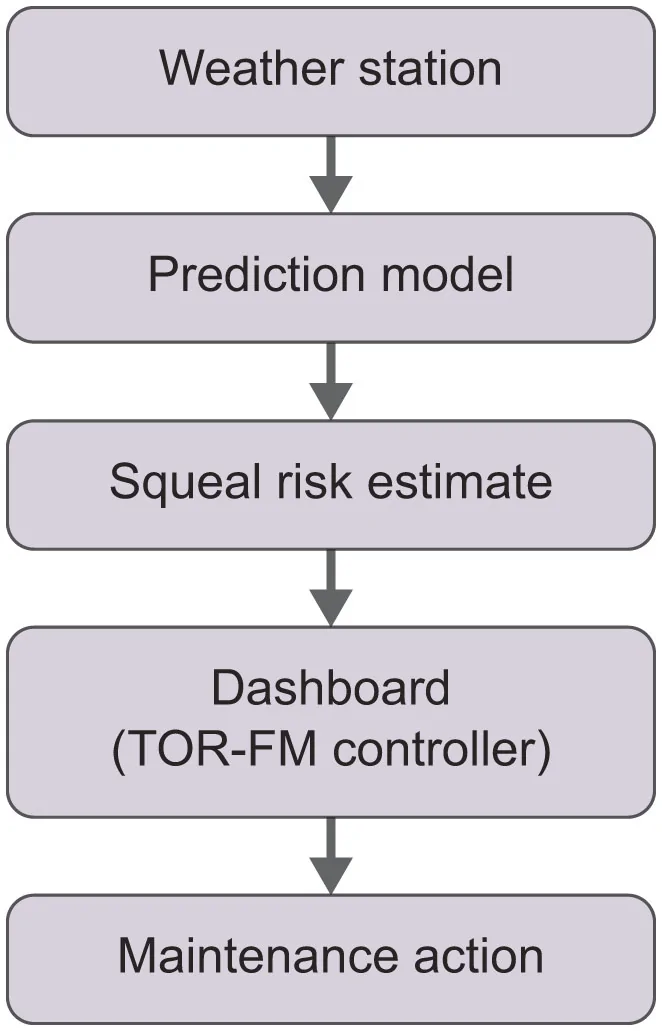
Conceptual diagram of operational implementation.

A potential implementation of the prediction model could consist of a weather station with rail temperature sensors and a prediction module integrated into a monitoring or TOR-FM system. Environmental measurements would be continuously measured and supplied to the prediction model, which would estimate the current probability of low-rail and high-rail squeal occurrence. The estimated probabilities could then be displayed on a dashboard interface as a decision support tool or integrated with TOR-FM systems to automatically adjust friction modifier application according to the predicted squeal risk. The conceptual diagram of proposed implementation would follow the steps shown in Figure 21. If used as decision support tool, the estimated squeal probabilities from the model should be calibrated to correspond to actual probabilities, as the direct predicted probabilities of the models are not to be interpreted as direct estimates of the probability of squeal occurrence.

Such implementation could support more targeted noise mitigation strategies by identifying periods with elevated squeal risk while reducing unnecessary TOR-FM application during low probability conditions. However, practical deployment would require additional validation under operational conditions and models would have to be validated at several locations or site-specific models should be used.

### 4.5 Limitations

A major limitation of the study is that the dataset was collected from a single railway curve, with its local climate, curve geometry, traffic characteristics and wheel and rail conditions. In addition, the curve was operated solely by a single train type. These factors influence squeal generation and the feature tendencies identified by the machine learning models. Therefore, the reported results should be considered site-specific and not as generalizable to other locations. Validation using data from multiple curves, climates, and trains would be required for generalisable results.

Another limitation is the risk for overfitting the model to the dataset during training. Although measures were taken to reduce overfitting, including chronological train-test splitting and time-series cross-validation during hyperparameter tuning, the risk of overfitting exist, especially regarding the limited number of positive samples. The reported performance should therefore be interpreted in the context of the measurement site, data collection period and chosen model hyperparameters. Larger datasets covering longer observation periods would improve the robustness of the models.

The classifier performances are highly influenced by the quality and representativeness of the training dataset. For squeal prediction, the used dataset was assumed to be of adequate quality, and potential inaccuracies or biases arising from data acquisition are not explicitly addressed. However, it is important to recognize that any imperfections of the dataset can significantly affect the generalization capability and predictive performance of the classifiers. Detailed discussion of the limitations regarding the dataset and data collection are provided in Toratti et al. (2026).

In this study a selection of machine learning algorithms were chosen and compared on a binary classification task. These represent probabilistic (GNB), linear combination (LR), ensemble (RF), and boosting (XGB) models to cover different types of machine learning algorithms. However, it is possible that alternative algorithms such as neural networks could yield a better predictive performance with possibly a trade-off in computational efficiency or robustness.

A notable limitation of the present study is also the absence of precipitation-related variables. Surface contamination and lubrication caused by rain, snow, and other environmental contaminants can significantly alter the wheel–rail contact conditions and are therefore expected to influence curve squeal occurrence. Variables such as recent precipitation, accumulated rainfall, snow cover and time since the last precipitation event could provide valuable information for characterizing these transient contact conditions. Precipitation variables were not included because the measurement system was not equipped with a rain sensor, and the nearest public weather station was located several kilometers from the investigated curve, making the available precipitation data not representative of the local conditions. Consequently, the developed models may not fully capture short-term changes in wheel–rail contact conditions associated with precipitation, which could partly explain the moderate predictive performance of the models. Although relative humidity and dew point may provide indirect information regarding moisture and rain, they cannot fully represent the effects of surface contamination and changing contact conditions. Future work should therefore incorporate direct precipitation measurements to improve prediction performance and better characterize the physical conditions leading to squeal.

## 5 Conclusions

This study investigated the predictability of railway curve squeal occurrence using environmental variables and machine learning models trained on a labeled dataset collected during a 19-month measurement campaign. Separate prediction models were developed for low-rail and high-rail squeal events, enabling rail-specific assessment of predictive performance and feature importances.

Among the evaluated algorithms, the tree-based models achieved generally the highest predictive performance. The results suggest that environmental variables contain useful predictive information regarding squeal occurrence, although the overall prediction performance is limited.

The models identified different feature importance patterns for low-rail squeal and high-rail squeal prediction, suggesting that the influence of environmental variables is not identical for the squeal types. These results indicate that treating low-rail and high-rail squeal as separate prediction tasks, rather than a single task, provides additional value for modeling squeal occurrence.

The operational assessment demonstrated the practical potential of the models for adaptive top-of-rail friction modifier (TOR-FM) control. The results indicate that substantial reductions in TOR-FM application rate could be achieved while maintaining high detection rates of squeal events, suggesting that environmental data based prediction may support targeted noise mitigation strategies.

The present study should be regarded as a proof-of-concept study with site-specific nature. The models were developed using data from a single railway curve with a specific geometry, operating condition, climate, and train type. Consequently, the results do not demonstrate generalisability to other locations. Future work should evaluate the approach at additional curves, climates, and traffic conditions, as well as investigate the inclusion of additional features such as precipitation-related variables.

In summary, the results show that environmental monitoring combined with machine learning methods can provide useful information regarding the risk of railway curve squeal occurrence. While the predictive performance is limited, the operational assessment showed promising results for application in predictive noise mitigation strategies.

## Figures and Tables

**Figure:**
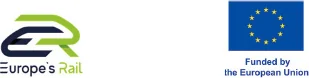


## Data Availability

The datasets presented in this article are not readily available because the dataset used in this study is not publicly available as it is part of ongoing research activities. Access to the data may be granted by the authors upon request. Requests to access the datasets should be directed to Leevi Toratti, leevi.toratti@ltu.se.
