# Supplementary material for: Railway curve squeal prediction using environmental variables
Source: Front Artif Intell. 2026 Aug 27;9:1887534. doi: 10.3389/frai.2026.1887534 (PMC13560889; doi:10.3389/frai.2026.1887534)

Appendix A. Feature distributions

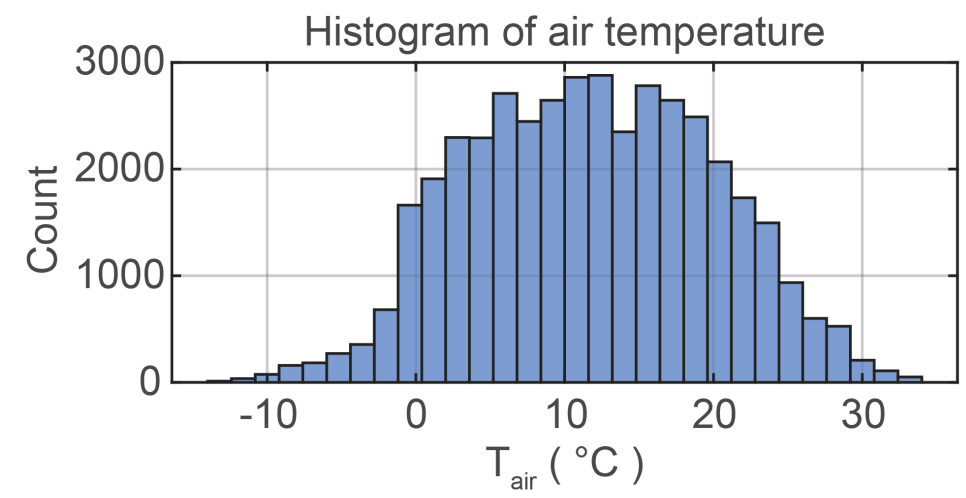

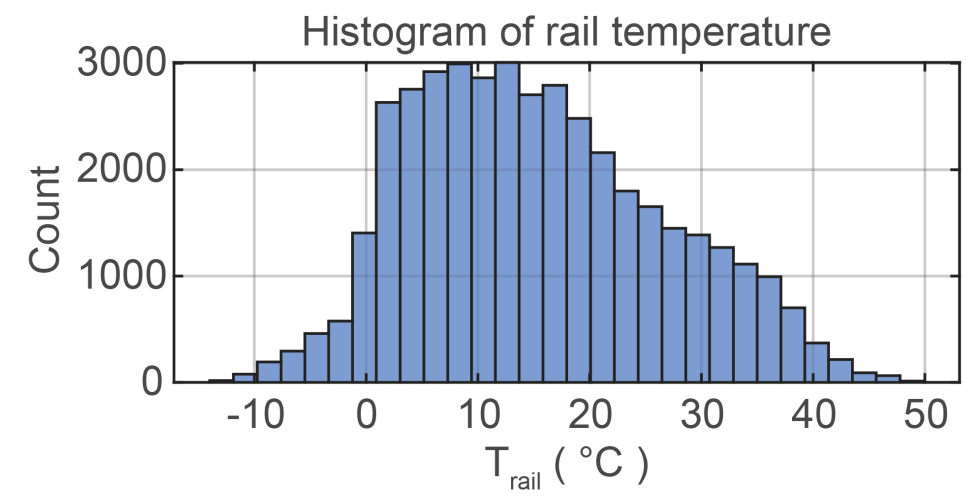

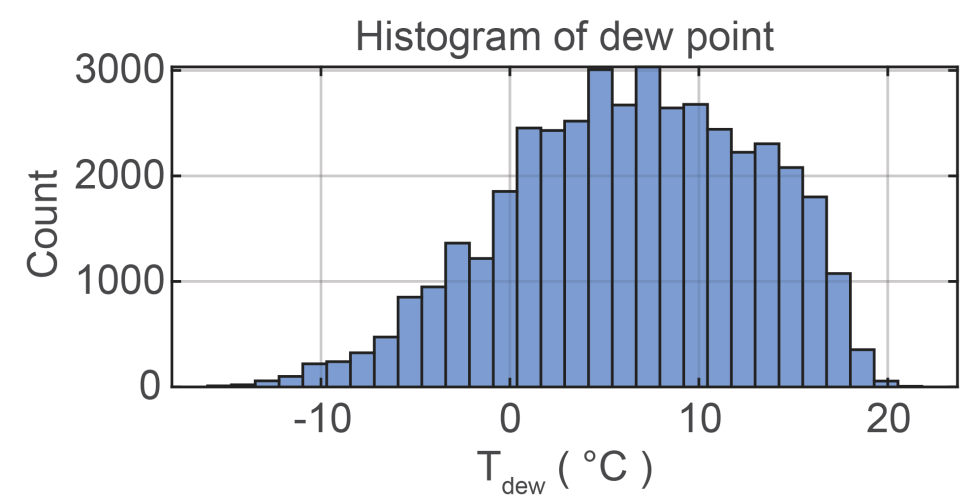

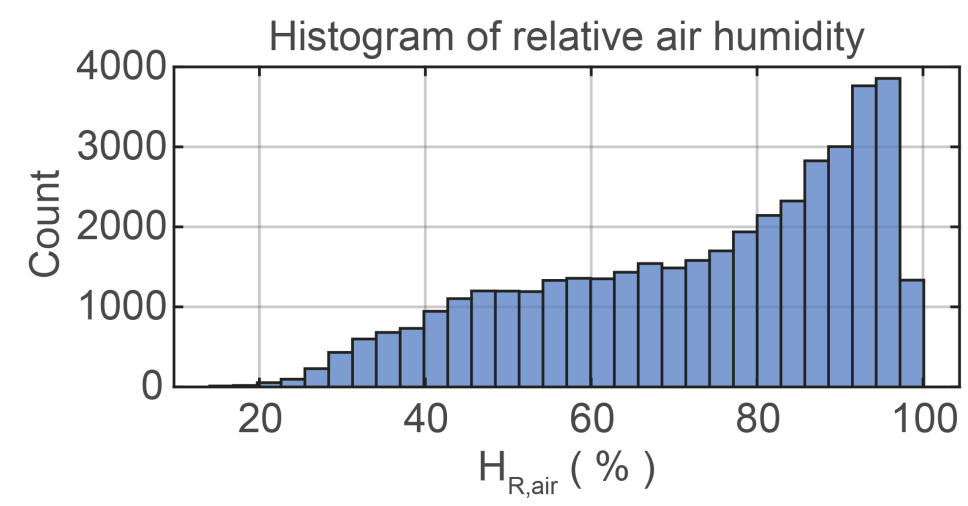

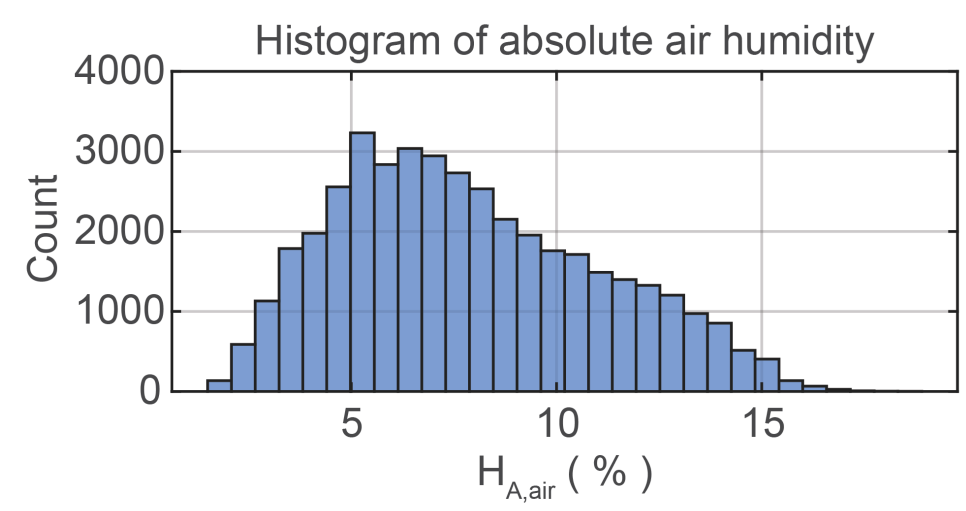

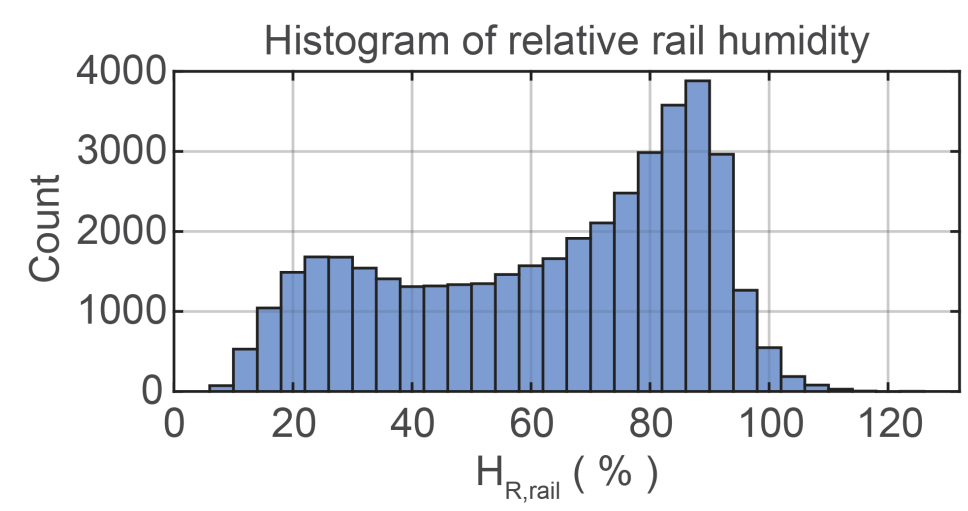

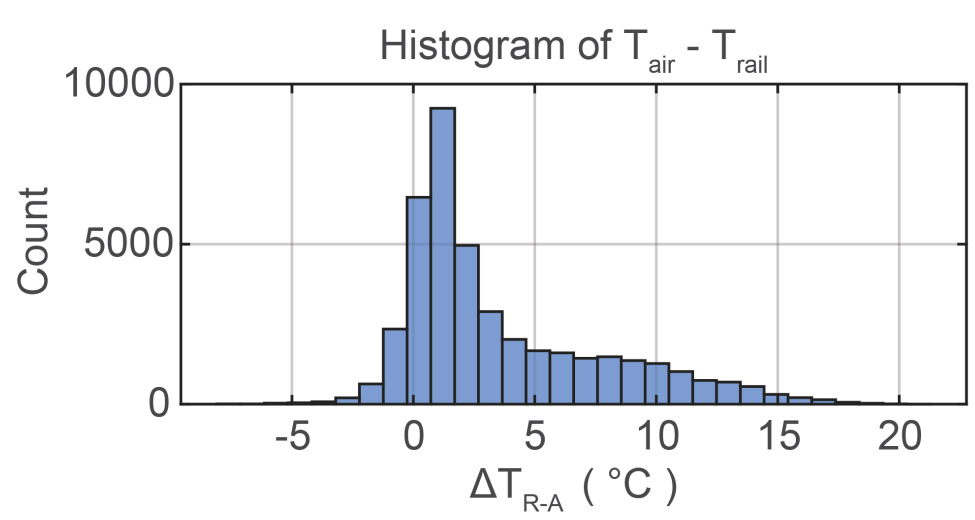

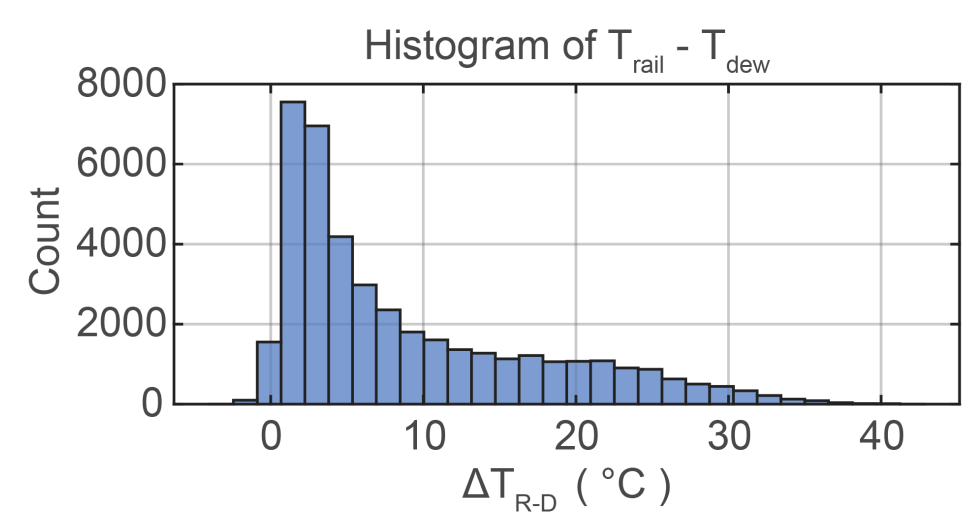

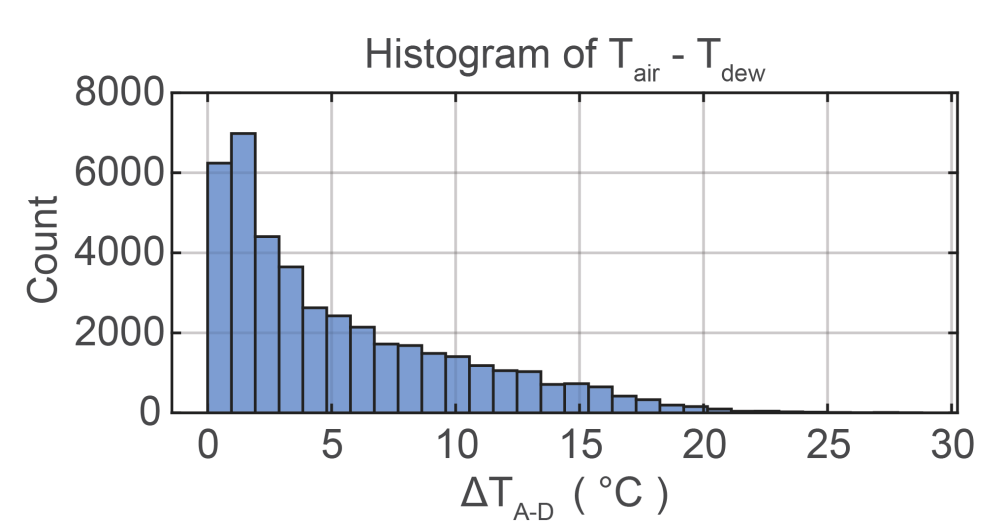

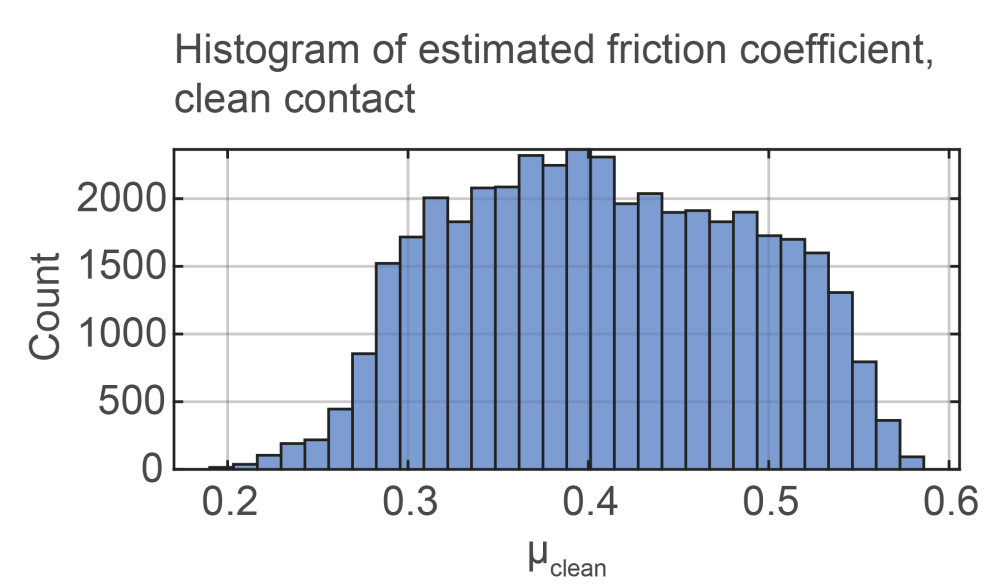

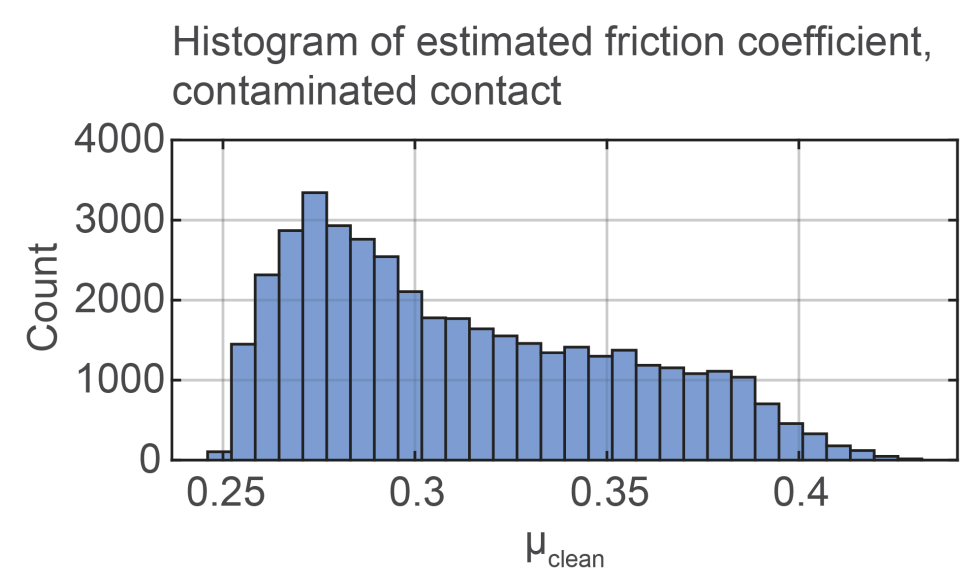

Supplement: Supplementary file 1 [file Data_Sheet_1.pdf]
