# Supplementary material for: Railway curve squeal prediction using environmental variables
Source: Front Artif Intell. 2026 Aug 27;9:1887534. doi: 10.3389/frai.2026.1887534 (PMC13560889; doi:10.3389/frai.2026.1887534)
Supplement: Supplementary file 2 [file Data_Sheet_2.pdf]

Appendix B. Hyperparameter grids

- LR
  - `penalty`: {L1, L2}
  - `C`: 20 numbers evenly spaced on a logarithmic scale from 0.0001 to 10000
- RF
  - `n_estimators`: {50, 100, 150, 200, 300, 400, 500}
  - `max_depth`: {3, 4, 5, 6, 7, 8}
  - `min_samples_split`: {1, 2, 3, 5, 10, 15, 20, 25, 30}
  - `min_samples_leaf`: {3, 5, 10, 20, 30, 40, 50, 60, 70}
- XGB
  - `n_estimators`: {50, 100, 150, 200, 250, 300, 400, 500}
  - `max_depth`: {2, 3, 4, 5, 6, 7, 8}
  - `learning_rate`: {0.001, 0.01, 0.03, 0.05, 0.1}
  - `gamma`: {0, 0.1, 0.3, 0.5, 1, 2, 4, 6, 8, 10}
